# All‐Inorganic Perovskite Quantum‐Dot Optical Neuromorphic Synapses for Near‐Sensor Colored Image Recognition

**DOI:** 10.1002/advs.202409933

**Published:** 2024-12-16

**Authors:** Yung‐Chi Yao, Chia‐Jung Lee, Yong‐Jun Chen, Jun‐Zhi Feng, Hongseok Oh, Chin‐Shan Lue, Jinn‐Kong Sheu, Ya‐Ju Lee

**Affiliations:** ^1^ Program on Key Materials Academy of Innovative Semiconductor and Sustainable Manufacturing (AISSM) National Cheng Kung University No. 1, University Road Tainan City 70101 Taiwan; ^2^ Department of Photonics National Cheng Kung University No. 1, University Road Tainan City 70101 Taiwan; ^3^ Department of Physics Department of Intelligent Semiconductors Soongsil University 369 Sangdo‐ro, Dongjak District Seoul 06978 South Korea; ^4^ Department of Physics National Cheng Kung University No. 1, University Road Tainan City 70101 Taiwan

**Keywords:** colored image recognition, CsPbBr_3_, near‐sensor computing, neuromorphic vision system, quantum dots

## Abstract

As the demand for the neuromorphic vision system in image recognition experiences rapid growth, it is imperative to develop advanced architectures capable of processing perceived data proximal to sensory terminals. This approach aims to reduce data movement between sensory and computing units, minimizing the need for data transfer and conversion at the sensor‐processor interface. Here, an optical neuromorphic synaptic (ONS) device is demonstrated by homogeneously integrating optical‐sensing and synaptic functionalities into a unified material platform, constructed exclusively by all‐inorganic perovskite CsPbBr_3_ quantum dots (QDs). The dual functionality of each unit within the ONS device, which can be operated as either an optical sensor or a synaptic device depending on applied electrical polarity, provides significant advantages over previous heterogeneous integration methods, particularly regarding material selection, structural compatibility, and device fabrication complexity. The ONS device exhibits distinct wavelength responses essential for emulating colored image recognition capability inherent in the human visual system. Additionally, the seamless integration of electronics and photonics within a unified material system establishes a novel paradigm for optical retrieval, enabling real‐time perception of the encoded status of the ONS device. These findings represent substantial advancements in near‐sensor computing platforms and open a new horizon for all‐inorganic perovskite optoelectronic technologies.

## Graphical abstract

This work demonstrates an optical neuromorphic synaptic (ONS) device by homogeneously integrating optical‐sensing and synaptic functionalities into a unified material platform, constructed exclusively by all‐inorganic perovskite CsPbBr_3_ quantum dots, to emulate the colored image recognition capabilities of the human visual system.

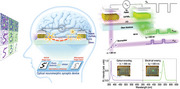

## 1 Introduction

Recent progress in artificial synaptic devices holds great promise for the development of neuromorphic vision systems, which aim to emulate the human brain's cognitive processes by integrating image sensors with computing units.^[^ ^1^, ^2^, ^3^, ^4^, ^5^ ^]^ These systems facilitate the real‐time acquisition, recognition, and classification of object images in response to outer visual stimuli. Central to these advancements is the artificial neural network (ANN),^[^ ^6^ ^]^ which serves as a vital conduit for bridging acquired analog data with subsequent digital computing units. Consequently, neuromorphic vision systems have found utility across diverse fields including autonomous vehicular technologies, intelligent manufacturing processes, and advanced healthcare systems.^[^ ^7^, ^8^, ^9^ ^]^ Traditionally, image sensors have been connected externally to memories and computing units following the von Neumann architecture,^[^ ^10^, ^11^ ^]^ owing to differences in their functionalities and fabrication technologies. However, this architecture leads to inefficiencies caused by extensive data conversion and transportation procedures. Sensing generally occurs in the noisy analog domain, resulting in large amounts of raw data at sensor terminals. These unprocessed analog data, often containing redundant background information, require conversion into digital signals through an analog‐to‐digital converter, before being stored in memory and subsequently transmitted to computing units for specific tasks such as image recognition or classification. The inherent inefficiencies of the von Neumann architecture pose significant challenges in energy consumption, temporal latency, and communication bandwidth, limiting the potential applications of neuromorphic vision systems. To address the von Neumann bottleneck, there is an urgent need for an enhanced architectural paradigm that features streamlined data transportation between sensor terminals and computing units.

To this end, significant attention has been devoted to the development of artificial optic‐neural networks by integrating photodetectors with synaptic devices such as 2D transistors^[^ ^12^, ^13^, ^14^ ^]^ or oxide memristors.^[^ ^15^, ^16^, ^17^, ^18^ ^]^ This integration strategy facilitates the direct conversion of analog information captured by the photodetectors into discernible conductance states within adjacent synaptic devices. Furthermore, these synaptic devices possess the capability to execute cognitive algorithms for image recognition, thereby endowing computational functionalities reminiscent of those demonstrated by the ANN. Termed “near‐sensor computing,” this paradigm leverages computational units situated in close proximity to sensory components.^[^ ^11^, ^19^ ^]^ By executing precise computational tasks directly at the sensor terminals, this framework enhances the synergy between sensors and computing units, consequently alleviating the need for extensive data transmission and reducing energy consumption. However, as outlined in the literature, prior approaches to near‐sensor computations often involve the integration of two separate devices comprised of vastly different material systems. Many constraints and challenges arise in such heterogeneous integration in terms of structural compactness, manufacturing compatibility, and synchronization of electrical and optical signal transmissions. For instance, a neuromorphic vision system described in ref. [15] employs a 1P‐1R (one photodiode and one memristor) crossbar configuration, achieved by vertically stacking an InGaAs photodiode onto a HfO_2_ resistive random‐access memory (RRAM). Such vertical integration of heterogeneous components poses a complex engineering challenge, requiring careful consideration to ensure the proper functionality of each device amidst different manufacturing processes. Similarly, an effort to emulate the image recognition capabilities of the human vision system, as proposed in ref. [12] introduces an optic‐neural synaptic device made by a van der Waals heterogeneous structure (h‐BN/WSe_2_). This structure requires the utilization of O_2_ plasma treatment to modulate the interface traps on h‐BN to induce synaptic dynamics. Again, the introduction of additional plasma treatment complicates the device fabrication, while the scalability of manufacturing is impeded by inherent limitations associated with the use of 2D materials. As a result, these prior demonstrates not only impose significant constraints on the material selection and compatibility, but markedly increase the complexity of the device fabrication. Therefore, there is a pressing need for an innovative architecture of near‐sensor computation, predicated upon one specific material that facilitates seamless integration between the sensory component and the synaptic device, to overcome these challenges.

In this study, we demonstrate an advanced ONS device that integrates optical‐sensing and synaptic functionalities into a unified material platform, exclusively utilizing all‐inorganic perovskite CsPbBr_3_ QDs. In contrast to previous studies that focused on either individual optoelectronic memory devices or complex integration processes involving diverse material systems,^[^ ^20^, ^21^, ^22^, ^23^ ^]^ our ONS device simplifies fabrication while demonstrating remarkable wavelength (color) response characteristics, making it suitable for emulating colored image recognition in the human visual system. Moreover, we demonstrate the real‐time perceptibility of the encoded status of the ONS device by synchronously detecting the optical power of photon wavelengths emitted upon entry or exit from the ONS device. These findings not only signify a significant advancement in the field of all‐inorganic perovskite optoelectronic technologies but also underline the potential for novel applications by fostering a cohesive synergy between electronics and photonics.

## 2 Results and Discussion

### 2.1 Design of the CsPbBr_3_ QD‐Based ONS Device

**Figure 1:**
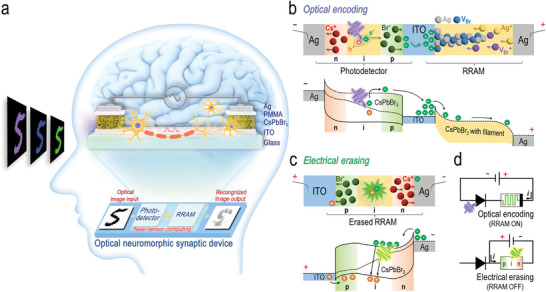
Implementation of near‐sensor computation utilizing the CsPbBr_3_ QD‐based ONS device to emulate the human visual system. a) Schematic of the neuromorphic vision system, comprising two nominally identical units (one serving as a photodetector and the other as an RRAM), seamlessly integrating to emulate colored image recognition in the human brain, and that of the block diagram illustrating the image recognition process within the 1P‐1R (near‐sensor) computing architecture. b,c) Schematics (top panels) elucidating ion migrations in the ONS and the RRAM devices, respectively, under applying either positive or negative electrical polarity to them. Their corresponding energy band diagrams are also plotted in the bottom panels of these figures. d) Equivalent circuit schematics illustrating the operational functionality of each unit constituted in the ONS device, under either positive (top panel) or negative (bottom panel) electrical polarity.

**Figure** **1**a illustrates the schematic configuration of our ONS device, which consists of two virtually identical units utilizing all‐inorganic perovskite materials (i.e., CsPbBr_3_ QDs) that seamlessly integrate each other (see Experimental Section), representing a notable advancement over conventional approaches in emulating synaptic dynamics. As detailed in our prior investigation,^[^ ^24^ ^]^ each perovskite unit demonstrates dual functionalities either as a p‐i‐n homojunction or an RRAM, completely switchable by altering the applied electrical polarity. Here, the p‐i‐n homojunction functions as a photodetector, while the interconnecting RRAM acts as a synaptic element, collectively constituting a monolithic 1P‐1R single‐pixel architecture. Although our ONS device with a horizontal 1P‐1R architecture provides lower integration density compared to the vertical design, it simplifies fabrication and enhances thermal management. The choice between vertical and horizontal configurations should be based on specific application requirements, including performance, density, thermal considerations, and fabrication capabilities. In order to achieve efficient modulation of conductance state representative of synaptic weight within the device, it is imperative to ensure appropriate resistance matching between the photodetector and the RRAM. Therefore, a comprehensive examination of the electrical characteristics in each unit was conducted prior to the commencement of operations (see Figures S1 and S2, Supporting Information). Additionally, the current‐voltage (*I‐V*) characteristics of both photodetector and RRAM devices show small variations between different units, demonstrating their reliability when integrated into the ONS device (see Figure S3, Supporting Information). Through careful manipulation of electrical polarity aided with a proper bias voltage applied to the ONS device, optical signals of incoming images (as colored number “5” denoted in the figure) captured by the photodetector can be effectively converted into electrical signals and stored in the RRAM, consequently enabling image memorization and pre‐processing such as noise reduction and contrast enhancement on the chip level (bottom plot, Figure 1a). This near‐sensor computing architecture significantly reduces the computational burden for subsequent image recognition tasks, as can be seen later in the article.

The design of the ONS device primarily relies on the orchestrated migration of ions in response to the applied electrical polarity, as depicted in Figure 1b,c. In the positive electrical polarity regime, a bias voltage is selectively applied to the top Ag electrode of the right unit, while the left unit remains grounded (top plot, Figure 1b). On the one hand, this setup induces the repulsion of cesium cations (Cs^+^) and bromide anions (Br^−^) toward the Ag electrode and the ITO, respectively, thereby establishing a p‐i‐n homojunction in the left unit. On the other hand, the migration of silver cations (Ag^+^) and bromide ion vacancy (V_Br_^+^) toward the ITO, along with subsequent reduction processes (as denoted by Ag^+^ + e^−^ → Ag and V_Br_^+^ + e^−^ → V_Br_), facilitates the formation of Ag and V_Br_ filaments in the right unit. While Coulomb attraction and repulsion forces continue to act on the charged ions within the perovskite material, they are minor in comparison to the forces generated by the applied electrical bias that drives ion migrations on a macroscopic scale (see Figure S4, Supporting Information). The energy‐band diagram corresponding to this state is depicted in the bottom plot of Figure 1b, which delineates a variety of energy levels comprising the conduction band edge (*E_C_*), the valence band edge (*E_V_*), and the Fermi level (*E_f_*) for each constituent element of the ONS device, including the Ag electrode, the CsPbBr_3_ QDs, and the ITO, determined through ultraviolet photoelectron spectroscopy (see Figure S5, Supporting Information). Upon light illumination on the left unit with photon energy surpassing the bandgap of the CsPbBr_3_ QDs, electron‐hole pairs are generated. The photogenerated electrons tend to drift toward the ITO, where they accumulate due to both the energy gradient of the *E_C_* and the predetermined electrical polarity applied to the ONS device. The accumulation of photogenerated electrons effectively increases the electrical potential of the ITO, consequently mitigating the discontinuity of the *E_C_* between the ITO and the CsPbBr_3_ QDs, accelerating filament formation of the RRAM on the right unit. In essence, incident optical signals intercepted by the p‐i‐n homojunction on the left unit result in the accumulation of photogenerated electrons within the ITO, and that in turn, enables the resistance modulation of the RRAM on the right unit via the formation of conductive filaments. This seamless conversion of optical information into distinct resistance states plays a pivotal role in emulating synaptic dynamics of the human visual system.

In the present study, we employ closed‐packed CsPbBr_3_ QDs synthesized by supersaturated recrystallization (see Experimental Section) as the active layers of the ONS device. Compared to another hot injection method typically used to synthesize CsPbBr_3_ QDs,^[^ ^25^ ^]^ the supersaturated recrystallization employed herein operates at a relatively lower temperature, reducing the overall thermal budget of the synthesis process. Additionally, the CsPbBr_3_ QDs have a diameter of ≈ 50–100 nm, much larger than the typical 10–15 nm size achieved using the hot‐injection method. This increased size results from the rapid transfer of Cs^+^, Pb^2+^, and Br^−^ ions from a soluble solvent to an insoluble one during the supersaturated recrystallization process, which significantly accelerates the growth of the QDs. Distinct fused interfaces between adjacent QDs can hence be observed (see Figure S6, Supporting Information). Despite the advantages of CsPbBr_3_ QDs, concerns about lead toxicity remain, especially for applications where environmental and human safety are crucial. Consequently, research is actively exploring lead‐free alternatives, such as tin (Sn)‐, bismuth (Bi)‐, and antimony (Sb)‐based perovskites,^[^ ^26^, ^27^, ^28^ ^]^ to mitigate these risks. However, lead‐based perovskites like CsPbBr_3_ continue to dominate the photoelectronic field due to their superior efficiency, photostability, and ease of synthesis. For lead‐free perovskite materials to gain widespread adoption, challenges related to stability, performance efficiency, and processability still need to be addressed. Absorption spectroscopy analysis conducted on these CsPbBr_3_ QDs reveals escalated optical absorption, particularly at shorter wavelengths (see Figure S7, Supporting Information). Therefore, exposure to light with shorter wavelengths leads to a discernible increment in the photocurrent of the p‐i‐n homojunction (see Figure S8, Supporting Information), suggesting potentially intensified accumulation of the photogenerated electrons within the ITO. This allows the adjustment of synaptic dynamic behaviors for the ONS device, primarily depending on the optical wavelength selected during light illumination. The ensuing synaptic plasticity under these conditions will be further investigated later in the article. A comprehensive analysis of photoresponse performances of our perovskite photodetector, including its responsivity (*R*_λ_), detectivity (D*), and external quantum efficiency (*EQE*), was also conducted (see Figure S9, Supporting Information). Conversely, when subjected to an opposing electrical polarity exceeding the RESET threshold of the RRAM on the right unit (top plot, Figure 1c), the previously encoded optical information is erased, as both Ag and V_Br_ filaments start diminishing owing to their associated oxidation processes.^[^ ^29^ ^]^ Intriguingly, with further escalation of the reversed bias voltage, the negative poling field induces repulsive forces on Cs^+^ cations and Br^−^ anions, causing them to migrate in diametrically opposite directions, and that again, leads to the reconstruction of the p‐i‐n homojunction. Upon exceeding its turn‐on voltage, the applied revised bias, now acting as a forward bias due to parallel alignment between the homojunction's polarity‐orientation with the external electric field, triggers light emissions.^[^ ^30^ ^]^ These emitted photons, possessing optical wavelength consistent with the bandgap of the CsPbBr_3_ QDs, stem from the radiative recombination of injected electron‐hole pairs occurring within the intrinsic region (bottom plot, Figure 1c). Figure 1d shows equivalent circuit schematics of the ONS device, elucidating the operational functionality of each unit, under either positive (top panel) or negative (bottom panel) electrical polarity. Departing from traditional all‐electrical approaches, the distinctive features observed herein provide an alternative for retrieving information optically.

### 2.2 Optical Perception of Encoded States in the CsPbBr_3_ QD‐Based ONS Device

**Figure 2:**
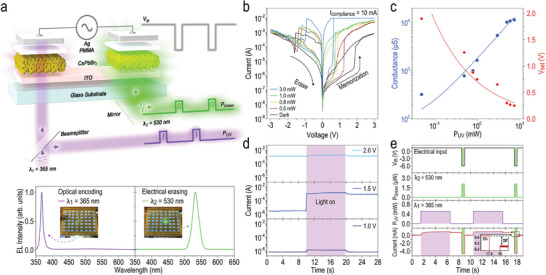
Light‐activated resistance switching in the CsPbBr_3_ QD‐based ONS device. a) Schematic illustration of the conceptual scheme in conjunction with the ONS device aimed at optically retrieving information. Within this scheme, the memorization process is activated through UV‐light illumination on the left unit, whereas the electrical erasing process is concomitant with green‐light emission from the right unit. Consequently, the encoded states of “write” or “erase” are discernible via synchronously detecting the optical power of UV (*λ_1_* = 365 nm) and green (*λ_2_* = 530 nm) emissions entering or exiting the ONS device. *EL* spectra, including UV‐light illumination on the left unit and green‐light emission from the right unit, are depicted in the bottom panels. The corresponding images of the ONS device when it operates under either optical encoding (left panel) or electrical erasing (right panel) condition are also inserted. The green glow visible in the left unit (left panel) comes from the photoluminescence emission of CsPbBr_3_ QDs when illuminated with UV light. b) Multiple memorization processes in the ONS device under UV‐light illuminations with various incident powers. Subsequent erase processes were conducted for each memorization state via negative voltage sweeps exclusively applied to the RRAM on the right unit. c) Conductance and SET voltage of the ONS device versus optical power of UV‐light illumination, extracted from light‐activated memorization processes. d) Dynamic evolution of transient current in response to UV‐light illumination under various positive voltages (+1.0, +1.5, and +2.0 V) applied to the ONS device. e) Transient response of the ONS device during the light‐activate SET and electrical RESET processes. A 0.5 mW UV‐LED was directed into the left unit of the ONS device for 5 s (the purple‐highlighted area), aided by a positive bias of +1.5 V, to set the device; while a reversed bias of ‐6 V was applied for 0.5 s to reset the ONS device, concomitant with green emission out from the right unit (the green‐highlighted area). Inset: enlarged time trace of the region with a reverse bias (−6 V, 0.5 s) fed into the ONS device, validating the resistive switching from LRS to HRS in the RRAM.

In this study, we also aim to explore the potential applications afforded by the unique ion migration properties inherent in the perovskite materials. Therefore, we present a conceptual framework that incorporates the ONS device as an innovative approach to achieving information retrieval, as illustrated in **Figure** **2**a. The schematic depicts the activation of the memorization process through UV‐light illumination on the left unit (, termed optical encoding), whereas the electrical erasing process is concomitant with green‐light emission on the right unit.

To avoid crosstalk signals from adjacent devices when measuring the ONS device, we focus the illuminated light source on the left unit (i.e., photodetector) from the bottom side, ensuring it covers an area smaller than the unit device that has a side length of ≈1.0 mm. This framework, unlike traditional electrical probing methods, enables parallel, non‐contact, and real‐time perception of encoded states—whether in the “write” or “erase” state—by synchronously detecting the optical power of photon wavelengths emitted upon entry (*λ_1_* = 365 nm) or exit (*λ_2_* = 530 nm) from the ONS device. This cohesive integration of electronics and photonics into a unified material system establishes a novel paradigm for the optical retrieval of encoded states. Electroluminescent (*EL*) spectra, encompassing both UV‐light illumination on the left unit and green‐light emission from the right unit, are also depicted in the figure, where corresponding images of the ONS device operating under either optical encoding (left panel) or electrical erasing (right panel) condition are included. Figure 2b shows a series of consecutive variations in the *I–V* characteristics throughout the memorization and erase processes in the ONS device, under varying incident powers of UV‐light illuminations directed toward the photodetector on the left unit. To conduct this measurement, positive voltage sweeps (0 V → +3 V → 0 V) were administered across the ONS device to implement a light‐activated SET (memorization) operation, while negative voltage sweeps (0 V → ‐3 V → 0 V) were exclusively applied to the RRAM on the right unit to enact a RESET (erase) operation. During the light‐activated memorization process, the *I‐V* characteristics of the ONS device exhibit similarities with those commonly observed in conventional RRAMs. They switch sharply in electrical resistance from a high‐resistance state (HRS) to a low‐resistance state (LRS), upon applying a positive voltage that exceeds the threshold required for SET operation. Conversely, during the erase process, the RRAM promptly reverts from LRS to HRS as a result of the negative voltage sweeping process, regardless of the prior incident power of UV‐light illumination on the device. Additionally, these *I–V* curves depict multiple memorization and erase processes, each correlated with distinguishable resistance states, significantly influenced by the level of incident power. This phenomenon is attributed to the heightened accumulation of photogenerated electrons within the ITO under higher incident power levels, leading to filament formations with increased conductance and lower threshold for SET operation (Figure 2c). The ability to modulate numerous resistance states holds paramount significance in synaptic devices, particularly in relation to potentiation and depression behaviors crucial for plasticity functionalities. In order to assess the feasibility of utilizing light to change the resistance state of the ONS device for directly encoding information, an investigation into the device's transient current in response to UV‐light illumination was conducted. Figure 2d illustrates the dynamic evolution of this response across a range of positive voltages (+1.0, +1.5, and +2.0 V) applied to the ONS device. Specifically, the left unit is subjected to UV‐light illumination at an incident power of 0.5 mW for a duration of 10 s (highlighted in purple, Figure 2d). It is noteworthy that the SET voltage of the ONS device under dark conditions is ≈1.9 V (in reference to the observation in Figure 2b), and the duration of UV‐light illumination must exceed 7 ms (or be less than 142 Hz) to effectively activate the optical encoding process in the ONS device (see Figure S10, Supporting Information). This frequency is higher than the 60 Hz flicker rate detectable by the human eye, making the high‐speed temporal resolution of the ONS device developed in this study well‐suited for emulating the human visual system. At a low bias operation of +1.0 V, the ONS device consistently maintains HRS even after UV‐light illumination, with only a slight increase in current due to the contribution of the photocurrent during illumination (bottom panel, Figure 2d). Upon ramping the bias to +1.5 V (middle panel, Figure 2d), a substantial surge in current, spanning over two orders of magnitude, is observed. This surge in current persists even after the cessation of UV‐light, confirming a transition from HRS to LRS. This observation provides compelling evidence supporting the feasibility of light‐activated resistance switching, validating the direct optical encoding into the ONS device. Finally, considering that the ONS device is already in the LRS under a bias of +2.0 V (top panel, Figure 2d), the induced photocurrent has negligible impact on altering the conductance state of the device. The experimental result presented in Figure 2e further illustrates the potential of optically encoding the digital state of the ONS device via UV‐light illumination, and subsequently electrically erasing the encoded data concomitant with green‐light emission. This approach realizes real‐time perception of the encoded status of the ONS device by using two separate optical detectors, to independently, measure the optical power of UV and green emissions entering and exiting the device. Note that in the present proof‐of‐concept study, the optical encoding demonstrated herein is not limited solely to the use of UV‐light. The selection of UV‐light was made based on its efficacy in yielding the highest photocurrent response from CsPbBr_3_ QDs within the spectral wavelength range available in our experimental setup. In terms of reliability performance (see Figure S11, Supporting Information), the perovskite RRAM demonstrates strong retention stability, with both the HRS and LRS states maintained for over 10^4^ s and an ON/OFF current ratio of ≈10^2^ (Figure S11a, Supporting Information). An endurance test was also conducted on the ONS device in pulse‐sweeping mode, where the HRS and LRS states were alternated through either electrical (Figure S11b, Supporting Information) or optical (Figure S11c, Supporting Information) activation. Despite minor fluctuations, the HRS/LRS ratio (≈ 10^2^) remains stable after more than 10^4^ sweep cycles. These results confirm the reliable and reproducible write/erase performance of the ONS device.

### 2.3 Synaptic Dynamics in the CsPbBr_3_ QD‐Based ONS Device

**Figure 3:**
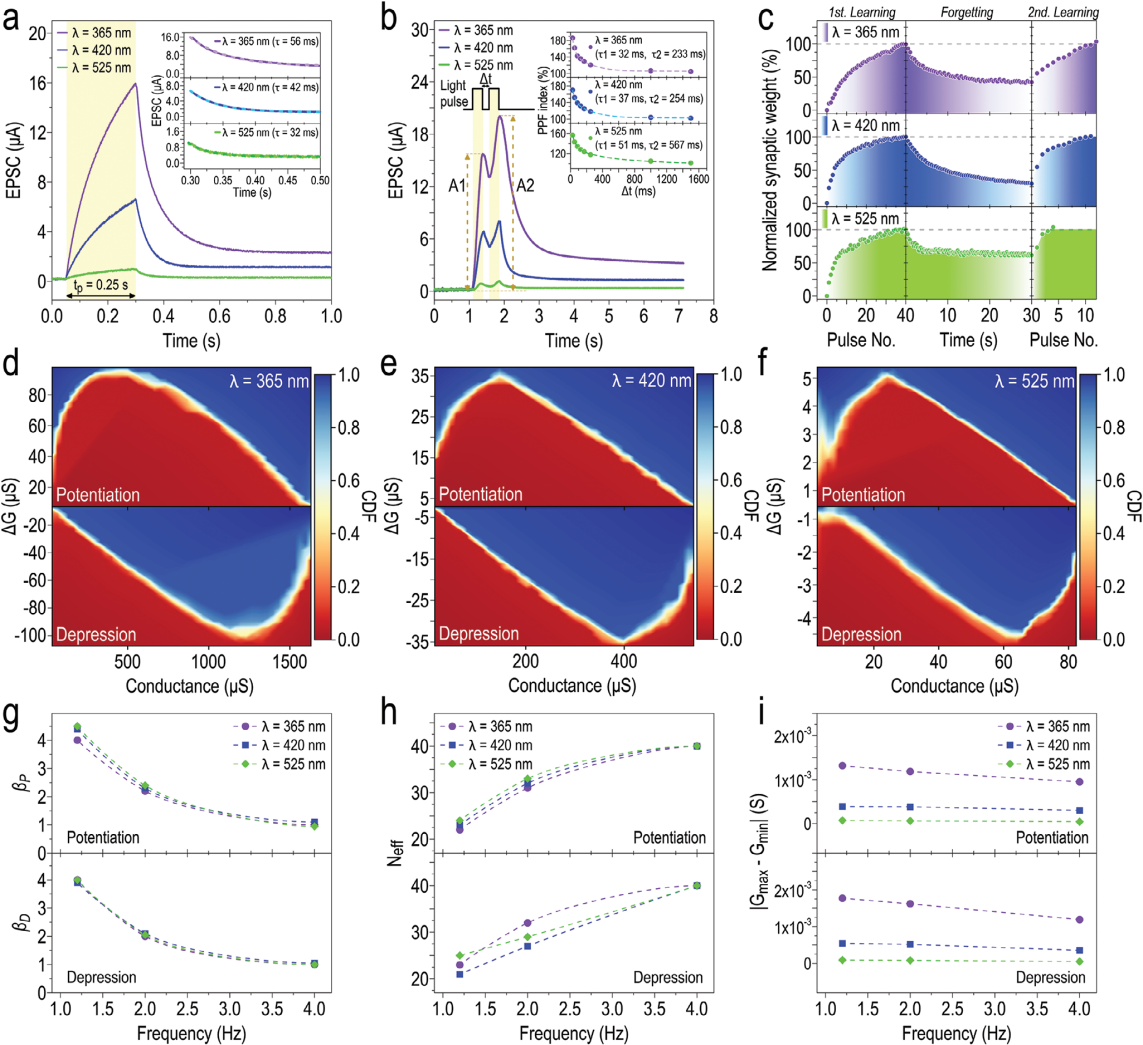
Synaptic plasticity characterization of CsPbBr_3_ QD‐based ONS device. a) EPSC response of the ONS device to a single light pulse (0.8 mW, 250 ms) illuminating on the left unit with different spectral wavelengths of *λ* = 365, 420, and 525 nm. Inset: magnified view of the time interval spanning from 0.3 to 0.5 s to observe the decay behavior of the EPSC, which is accurately modeled by the single exponential function (dashed line). b) EPSC variation triggered by two successively applied light pulses to the ONS device (0.8 mW, pulse width 250 ms, pulse interval 250 ms) across three spectral wavelengths of *λ* = 365, 420, and 525 nm. Inset: PPF index variation as a function of pulse interval (∆*t*) for three spectral wavelengths of interest. All of them can be suitably characterized by the double exponential function (dashed line), where *τ*_1_ and *τ*_2_, denoting the characteristic relaxation times for the rapid and slow decay phases, are also summarized within the corresponding figures. c) Emulation of knowledge acquisition in the ONS device, including learning, forgetting, and relearning processes, triggered by repetitive training light pulses with different spectral wavelengths. Conductance change (∆*G*) versus conductance (*G*) plot of the cumulative distribution function (CDF) for the ONS device under various spectral wavelengths of light stimuli of d) *λ* = 365 nm, e) *λ* = 420 nm, and f) *λ* = 525 nm. The ONS device's g) nonlinearity magnitude (*β*), h) the number of effective conductance states (*N*_eff_), and i) the conductance disparity (|*G*_max_ – *G*_min_|) versus the frequency of stimuli pulses (*f*) applied to the device, for three different spectral wavelengths (*λ* = 365, 420, and 525 nm) and for both potentiation (upper panel) and depression (bottom panel) processes.

Until now, we have observed the ONS device can transit into a high conductance state in response to light stimulation, a state that persists beyond the cessation of illumination. Specifically, the optical input perceived by the photodetector on the left unit undergoes transformation into electrical signals, effectively assuming the function of presynaptic stimuli. These elicited electrical signals are subsequently relayed to the RRAM component on the right unit, facilitating the emulation of synaptic dynamics. This unique synaptic behavior, modulable for either potentiation or depression using appropriate optical and electrical pulse triggers, holds significant implications in the neuromorphic computing realm. Next, we demonstrate that the ONS device serves as a fundamental artificial synaptic unit analogous to those found in the human visual system. In this circumstance, the external optical pulse operates as the presynaptic stimulus, evoking postsynaptic responses discernible through the measurement of electrical currents traversing the device. The synaptic plasticity exhibited by the ONS device, inclusive of excitatory postsynaptic current (EPSC), paired‐pulse facilitation (PPF), and long‐term potentiation/depression (LTP/LTD), is systematically examined in correlation with diverse spectral wavelengths of optical stimuli. To more accurately simulate the human visual system, it is crucial to assess our ONS device's performance under different stimulus conditions, including emission wavelength, pulse duration, frequency, and repetition rate. **Figure** **3**a depicts the EPSC characteristics of the ONS device under various spectral wavelengths of light stimuli (*λ* = 365, 420, and 525 nm). Throughout the measurements, light stimulus remained constant in both duration (*t*_p_ = 0.25 s) and optical power (*P* = 0.8 mW), while a forward bias of +0.1 V was applied to the ONS device. Under the light stimulus, the EPSC exhibits a progressive increase, succeeded by an exponential decay upon removal of light. Moreover, the application of shorter spectral wavelengths of light results in a heightened augmentation of the EPSC, escalating from 0.98 µA (*λ* = 525 nm) to 6.59 µA (*λ* = 420 nm), and further to 15.96 µA (*λ* = 365 nm). This augmentation is accompanied by a notable prolongation in the decay time, progressing from *τ* = 32 ms to *τ* = 42 ms, and subsequently to *τ* = 56 ms (inset, Figure 3a). These findings primarily originate from the generation of a greater number of photogenerated carriers within the device when exposed to light stimuli with shorter wavelengths.^[^ ^3^, ^31^ ^]^ In Figure 3b, we present the PPF dynamics of the ONS device, a type of short‐term plasticity (STP) arising from the application of two closely spaced light pulses, with a temporal interval (Δ*t*) of 250 ms in this experimental setup, across three spectral wavelengths of interest (*λ* = 365, 420, and 525 nm). It is observed that the peak intensity of the EPSC elicited by the second light pulse (*A_2_*) surpasses that evoked by the initial light pulse (*A_1_*). This phenomenon is attributed to the presence of residual photogenerated carriers remaining after the initial light pulse. Prior to attaining an equilibrium state via recombination or diffusion processes, these residual carriers pile up alongside those generated by the subsequent light pulse, thereby augmenting the EPSC amplitude, as well as the synaptic weight of the device.^[^ ^32^ ^]^ The corresponding PPF indices, defined as the ratio of the amplitudes between *A_2_* and *A_1_* (i.e., PPF = (*A*_2_/*A*_1_) × 100%), are depicted as a function of Δ*t* in the inset of Figure 3b. At Δ*t* = 25 ms, a maximum PPF index is attained, exhibiting values ranging from 156% (*λ* = 525 nm) to 170% (*λ* = 420 nm), and up to 186% (*λ* = 325 nm), aligning closely with reported values of artificial synapses in the literature.^[^ ^33^ ^]^ As Δ*t* increases, the observed PPF index gradually declines, displaying a decay pattern reminiscent of the kinetics observed in biological synapses, which can be aptly described by the double exponential function as expressed by:^[^ ^34^ ^]^

(1) $$PPF\;index=C_{0}+C_{1}\exp\left(-\frac{\Delta t}{\tau_{1}}\right)+C_{2}\exp\left(-\frac{\Delta t}{\tau_{2}}\right)$$

where *C*_0_, *C*_1_, and *C*_2_ represent the initial facilitation magnitudes, and *τ*_1_ and *τ*_2_ denote the characteristic relaxation times for the rapid and slow decay phases, respectively. Irrespective of the spectral wavelength of the light pulse, it was found that the extracted value of *τ*_1_ is approximately one order of magnitude faster than that of *τ*_2_, a phenomenon commonly observed in the decay behavior of biological synapses.^[^ ^35^ ^]^ In addition to the STP behaviors observed above, the LTP action emerges as another important metric for evaluating the efficacy and operational integrity of artificial synapses. The LTP action of the ONS device is triggered with repetitive training light pulses, mimicking the cyclic nature of learning, forgetting, and relearning intrinsic to the human visual system during knowledge acquisition. To emulate such cognitive dynamics experienced in the ONS device, a sequence of 40 consecutive light pulses (*P* = 0.8 mW, *t*_p_ = 0.25s, and Δ*t* = 0.25 s) with varying spectral wavelengths (*λ* = 365, 420, and 525 nm) is implemented. This sequence activates the initial phase of learning, followed by a pause of 30 s to simulate the forgetting phase, and succeeded by the commencement of the second learning phase using identical light pulse stimulations as the initial phase. Throughout these cyclic processes, where a positive bias of +0.1 V is applied to the ONS device to monitor the variation in EPSC, the corresponding synaptic weight is estimated and graphically depicted against the number of light pulses (Figure 3c). Accordingly, the normalized synaptic weight consistently increases with successive light pulse stimulations, indicative of enhanced memory consolidation during the initial learning phase, regardless of spectral wavelengths utilized in light pulse stimulations. Synaptic weight then declines gradually following the cessation of light stimuli, representing the decay in memory retention. Further stimulations using an additional sequence of light pulses can effectively restore the lost synaptic weight during the forgetting phase, and that needs only fewer light pulses to attain the same weight level as previously achieved in the initial learning phase.

Figure 3d–f shows the conductance change (∆*G*) versus conductance (*G*) plot of the cumulative distribution function (CDF) for the ONS device under light stimuli with various spectral wavelengths of *λ* = 365, 420, and 525 nm. The statistical model underlying these plots is derived from conductance updates recorded over 10 cycles of LTP/LTD synaptic behaviors for each spectral wavelength. These plots demonstrate the probability of achieving specific ∆*G* values for a given *G* value,^[^ ^36^ ^]^ including both potentiation (upper panel) and depression (bottom panel) processes. Here shall be addressed that the depression process is triggered by applying electrical pulses of −2 V, with on and off times of 0.25 s each, to the ONS device. It indicates the intrinsic nonvolatile synaptic plasticity present in our ONS device, with the application of negative electrical pulses being essential for depressing the potentiated device by disrupting conductive filaments established in the RRAM on the right unit. Accordingly, the CDF responses across different spectral wavelengths exhibit similar distribution profiles, indicating a consistent nonlinearity feature. During the potentiation process, the ∆*G* change corresponding to a CDF value of 0.5 (white region) exhibits a steep increase with the *G* value, reaching a saturation point before gradually declining. This observation suggests that at lower conductance levels, a small number of light pulses can induce a significant ∆*G* change in the ONS device. In contrast, additional light pulses result in a diminished ∆*G*, when the device operates in the middle or high conductance ranges. Similarly, in the depression process, a few electrical pulses rapidly induce a significant ∆*G* change while maintaining the ONS device in the high conductance state. As the number of electrical pulses increases, the ∆*G* change diminishes, and the device's conductance decreases toward the middle or low conductance range. These results offer valuable insights into the synaptic plasticity of the ONS device under light stimuli with varying spectral wavelengths, illustrating the influence of the number of light pulses in modulating the nonlinear characteristics of conductance changes. To quantitatively evaluate the linearity of synaptic dynamics in response to variations in the spectral wavelength of light stimuli, we estimate the nonlinearity (*β*) and the number of effective conductance states (*N*_eff_) of the ONS device (see Experimental Section). Both parameters are critical in determining the recognition accuracy of synaptic devices. A higher *β* value indicates greater nonlinearity, which makes it harder to precisely control conductance changes in response to input pulses. Furthermore, as nonlinearity increases, achieving a sufficiently high *N*_eff_ becomes more difficult, further complicating the tuning of the device's synaptic weights (see Figure S12, Supporting Information). At a given frequency of input pulses (*f*), the ONS device demonstrates a consistent *β* value regardless of variations in spectral wavelength. This phenomenon of nonlinear potentiation (*β_P_*)/depression (*β_D_*) manifests a gradual decline from 4.5/4.0 to 2.4/2.0, further diminishing to 1.1/1.0, with increasing *f* from 1 to 4 Hz (Figure 3g). Conversely, the corresponding *N*_eff_ exhibits an inverse correlation with the increment of *f*, albeit remaining relatively stable at a specified *f* despite the different spectral wavelength conditions (Figure 3h). As depicted in Figure 3i, the conductance disparity between the maximum (*G*_max_) and minimum (*G*_min_) values extracted from both LTP and LTD synaptic characteristics ranges in different levels, largely determined by the spectral wavelength of light stimuli. As a result, given the fixed presynaptic stimulus frequency, the resultant synaptic weights are adaptable in line with the spectral wavelengths of light stimuli applied to the ONS device, while maintaining a nearly identical nonlinearity characterized by comparable *β* and *N*_eff_ values. Overall, we found that i) the changes in conductance associated with the LTP/LTD synaptic characteristics vary significantly depending on the illuminating wavelength, and ii) the key parameters derived from these LTP/LTD characteristics, such as *β* and *N*_eff_, which strongly impact pattern recognition accuracy, remain largely unaffected by the wavelength of light. These unique properties allow our ONS device to maintain consistent learning patterns across various spectral wavelengths (see Figure 3c) and to achieve similar accuracy in recognizing UV, blue, and green MNIST handwritten digits over multiple training epochs (as further discussed later in the article), even though the device exhibits the highest photoresponsivity under UV‐light illumination compared to other visible‐light wavelengths.

### 2.4 Colored Image Recognition in Using the CsPbBr_3_ QD‐Based ONS Device

**Figure 4:**
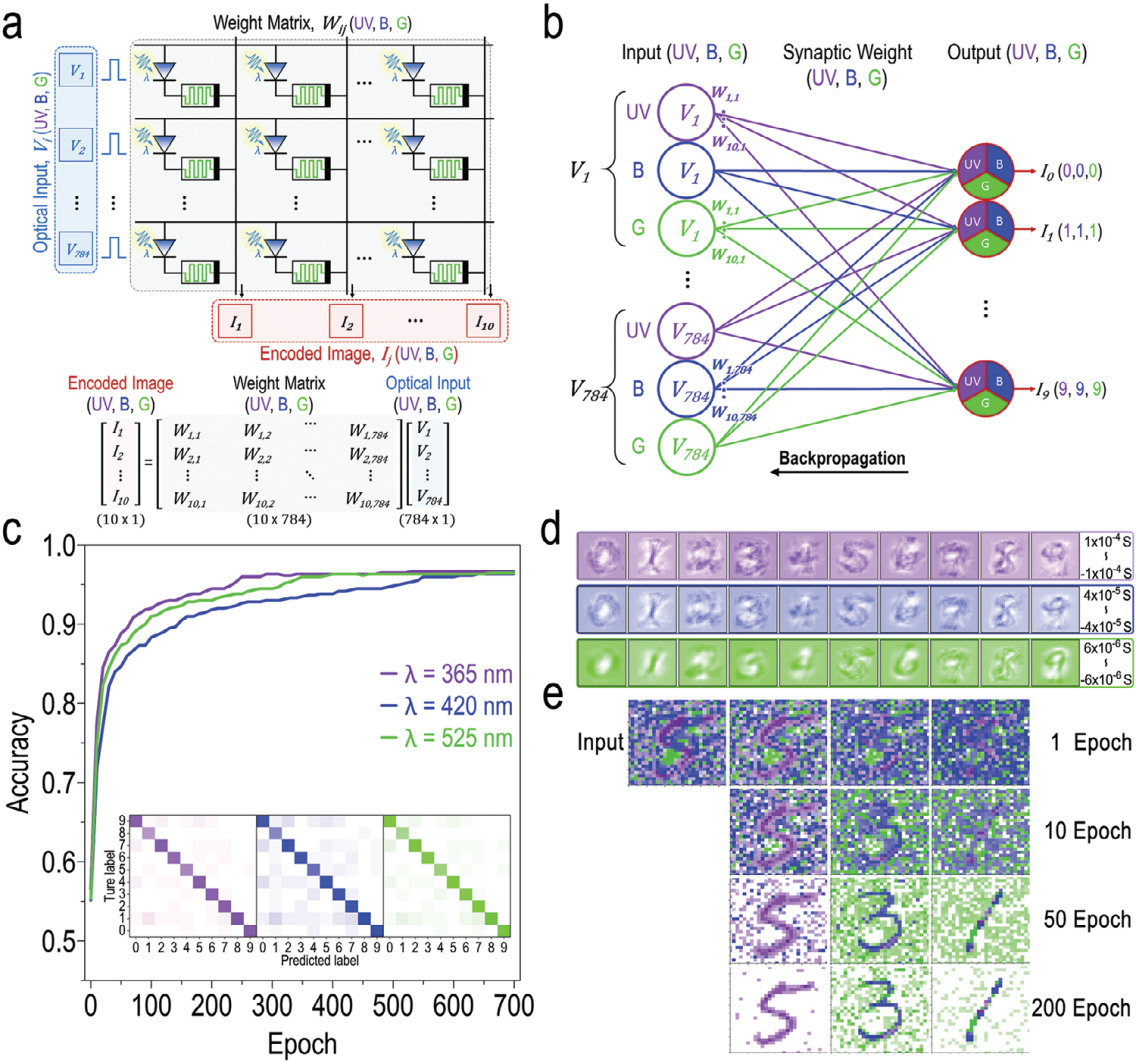
Colored and color‐mixed image recognition by using the CsPbBr_3_ QD‐based ONS device. a) Schematic illustration of the multiply‐accumulate operation of the neuromorphic encoding process using a 2D 28 × 28 array composed of the ONS devices. Corresponding matrix‐vector multiplication (bottom panel) is depicted with parameters of input optical signal *V_i_* (UV,B,G), synaptic weight *W_ij_* (UV,B,G), and output current signal *I_j_* (UV,B,G). b) Single‐layer artificial neural network illustrating the computational processes of using our ONS devices for colored image recognition. c) Recognition accuracy of the UV/B/G colored MNIST handwritten digits as a function of the number of training epochs. Insets: Confusion matrices of classification results for UV/B/G colored MNIST handwritten digits after 700 training epochs. d) Reconstructed mapping images of synaptic weights in accordance with ultraviolet (upper panel), blue (middle panel), and green (bottom panel) MNIST patterns after 700 training epochs. e) Encoded image (upper left) consisting of MNIST handwritten digits mixed with UV/B/G colored numerals, including 5 (UV), 3 (blue), and 1 (green), and intentionally introduced background noise. Classification results, obtained by feeding the encoded image into the trained neural network across various training epochs of 1, 10, 50, and 200 times, demonstrate distinct recognition capability for the MNIST handwritten digits in UV, blue, and green colors.

To realize neuromorphic vision systems, we utilize a conventional crossbar architecture employing the parameters extracted from the ONS device and a single‐layer artificial neural network. The image data is encoded within each ONS device in a matrix configuration, enabling simultaneous storage and computational processes required for the execution of colored image recognition tasks. **Figure** **4**a illustrates the multiply‐accumulate operation of the neuromorphic encoding process wherein image data is constructed by a 2D 28 × 28 array composed of the ONS devices. It is noteworthy that the color discrimination of image data, including ultraviolet (UV), blue (B), and green (G) hues, is achieved by applying distinct intensity thresholds to each color channel. These input optical signals, *V_i_* (UV,B,G), are then applied to the crossbar column as vectorized electrical signals. Each ONS device within the 2D array responds differently to the spectral wavelengths of UV, B, and G light, thereby inducing varying synaptic weights of *W_ij_* (UV,B,G), as discussed earlier in Figure 3. The corresponding matrix‐vector multiplication of the 28 × 28 ONS device array is depicted in the bottom panel of Figure 4a, in which the input optical signals of *V_i_* (UV,B,G) are applied at the array rows, resulting in the output current signals of *I_j_* (UV,B,G) in accordance with the Kirchhoff's and Ohm's laws, expressed as $I_{j}\left(UV,B,G\right)=\sum_{i}W_{ij}\left(UV,B,G\right)V_{i}\left(UV,B,G\right)$.^[^ ^37^ ^]^ Figure 4b shows a single‐layer artificial neural network that illustrates the computational processes of using our ONS devices for colored image recognition. The input layer of this network comprises 3 neurons, denoted as *V_i_* (UV,B,G), which simulate the variety of colors perceived by the human visual system. Synaptic connections are appropriately established in alignment with the optical‐sensing functionality, utilizing color‐dependent synaptic weights, *W_ij_* (UV,B,G), to ensure accurate colored image recognition. Each input neuron of *V_i_* (UV,B,G) is fully connected to 10 dedicated classifying output neurons, representing UV/B/G colored numerical images spanning from “0” to “9”. These connections are made by individual synaptic weights, *W_ij_* (UV,B,G), that are expressed as color lines to present their LTP/LTD characteristics in different UV/B/G conductance ranges. Whenever a colored image is inputted to the neural network, each *W_ij_* (UV,B,G) is appropriately updated by using the backpropagation algorithm to extract the optimum vectors of synaptic weights.^[^ ^38^ ^]^ To train the neural network, a total of 60000 training datasets from the MNIST database of handwritten digits were utilized, but a modification was implemented by setting different intensity thresholds to separate UV/B/G hues. Each dataset comprised images with dimensions of 28 × 28 pixels, including UV, B, and G colored images, covering digits from “0” to “9”. This preprocessing resulted in a total of 180000 colored images for training. For each color category in the training datasets, 600 different training images were randomly selected and applied to the networks at every training epoch. The training process was completed over 700 epochs. During each epoch, another 10000 randomly ordered images not included in the training process were used for testing to estimate the image recognition accuracy.

Accordingly, the accuracy rate for all UV/B/G colored MNIST handwritten digits exhibits a rapid increase with the progression of training epochs, surpassing 90% after the 200^th^ epoch and then reaching a plateau (Figure 4c). Even with further training epochs, however, the recognition accuracy for all three UV/B/G colored MNIST digits does not improve. Besides, the confusion matrix for recognition accuracy of each colored pattern after the 700^th^ epoch indicates that the inferred output numbers for each row align closely with the desired output numbers for each column (Insets, Figure 4c). This correspondence signifies accurate inference for each colored digit from “0” to “9”. These findings together validate the efficacy of using our ONS device as a critical component in neuromorphic vision systems for colored image recognition. The reconstructed mapping images of synaptic weights in accordance with ultraviolet (top panel), blue (middle panel), and green (bottom panel) MNIST patterns following 700 training epochs, provide further evidence of effective weight optimization (Figure 4d), where the synaptic weights created for numerical digits, represented in various colors, demonstrate comparable recognition irrespective of variations in their conductance values. Figure 4e presents the encoded image (upper left), which consists of MNIST handwritten digits mixed by UV/B/G colored numerals, including 5 (UV), 3 (blue), and 1 (green). Deliberate background noise has been incorporated into the image to enhance complexity. This encoded image is subsequently processed by the trained neural network, which employs optimized weight allocations to classify the numerical digits based on their color features. As a result of the distinct synaptic weights that respond to various spectral wavelengths of light stimuli and the progressive increase in training epochs, the neural network developed using our ONS devices is capable of accurately classifying the encoded digits as well as differentiating their constituent colors. This functionality effectively emulates the visual processing capabilities of the human brain.

## 3 Conclusion

In conclusion, we demonstrate an all‐perovskite‐based ONS device with optical‐sensing and synaptic dual‐functionality through the seamless integration of two nominally identical units, each leveraging the coordinated migration of ions in response to the applied electrical polarity across the device. Optical signals intercepted by the left unit induce the accumulation of photogenerated electrons within the ITO layer. Consequently, synaptic weight modulation occurs in the other unit through the formation of conductive filaments, mimicking the synaptic dynamics observed in the human visual system. The ONS device enables a parallel, non‐contact, and real‐time perception of encoded states, characterized by the detection of optical power at specific photon wavelengths (*λ_1_* = 365 nm for “write” state and *λ_2_* = 530 nm for “erase” state). Furthermore, our ONS device exhibits a variety of synaptic behaviors, including EPSC, PPF, LTP, and LTD, with synaptic weights adaptable to different spectral wavelengths of light stimuli while maintaining consistent nonlinearity, as indicated by similar *β* and *N*_eff_ values. We further simulate the multiply‐accumulate operation of the neuromorphic encoding process by using a 2D 28 × 28 ONS device array to emulate the colored image recognition capability of the human visual system. After 200 training epochs, the system achieves over 90% accuracy in classifying UV/B/G colored MNIST handwritten digits. The results underscore the potential of cohesive integration within a unified material platform in advancing neuromorphic computational research, particularly in complex image recognition tasks with integrated sensing and training functionalities.

## 4 Experimental Section

### Fabrication of the ONS Device

In this study, we developed the ONS device, consisting of two identical units fabricated on a glass substrate featuring indium tin oxide (ITO) rectangular pads. These ITO pads, with a thickness of 250 nm and dimensions of 4.0 mm × 7.0 mm, were formed using radio frequency (RF) magnetron sputtering through a shadow mask. Despite the spatial separation of ≈1.0 mm between the mesa areas of the left and right units, they were electrically connected in series due to the low resistivity of the ITO (≈4.8 × 10^−4^ Ω∙cm). The active layer for each unit was created by spin‐coating close‐packed CsPbBr_3_ QDs, synthesized via the supersaturated recrystallization method at room temperature, onto the ITO pads at 3000 rpm for 30 s. This process resulted in a quasi‐continuous film with a thickness of ≈800 nm. Subsequently, a polymethyl methacrylate (PMMA) protection layer was applied over the CsPbBr_3_ QDs at the same spin speed. A 100‐nm‐thick square‐patterned silver (Ag) electrode, with a side length of ≈1.0 mm, was then deposited on the PMMA layer using RF magnetron sputtering. This Ag electrode is typically employed to enhance the RRAM's ON/OFF ratio. During the fabrication process, both the top Ag (100 nm) and bottom ITO (250 nm) electrodes were deposited using an RF sputtering system under a background chamber pressure of ≈1.0 × 10^−6^ torr. The working pressure and sputtering power were maintained at ≈8.0 × 10^−3^ torr and 50 W, respectively. Argon gas was introduced into the sputtering chamber at flow rates of 6.0 sccm for Ag and 10.0 sccm for ITO during the deposition of the films. The deposition rate for both Ag and ITO films was ≈4.0 Å s^−1^.

### Synthesis of CsPbBr_3_ QDs

In this study, CsPbBr₃ QDs were synthesized via the supersaturated recrystallization method. Initially, lead (II) bromide (PbBr_2_, 0.1845 g) and cesium bromide (CsBr, 0.1415 g) powders were dissolved in dimethyl sulfoxide (DMSO, 5 mL) to prepare the precursor solution. Subsequently, oleic acid (OA, 0.5 mL), oleylamine (OAm, 0.25 mL), and hydrogen bromide (HBr, 5 µL) were added to the precursor solution to enhance its stability. The solution was maintained under continuous stirring at a rate of 1000 rpm for a duration of 1 h. The resultant mixed precursor solution was then introduced into toluene (240 mL) to initiate the supersaturated recrystallization process necessary for the synthesis of the CsPbBr₃ QDs. Following this, the entire solution underwent centrifugation at 3000 rpm for 5 s. Finally, the supernatant was carefully collected using a pipette to remove larger particles. All procedures were conducted at room temperature. The morphology, crystallinity, and homogeneity of the synthesized CsPbBr_3_ QDs were characterized using X‐ray diffraction (XRD) and transmission electron microscopy (TEM) measurements (see Figure S6, Supporting Information).

### Analysis of Nonlinearity and Number of Effective Conductance States

In the context of synaptic devices, the parameter *β* quantifies the degree of nonlinearity in conductance (*G*) as a function of external stimuli. The effective number of conductance states (*N*_eff_) denotes the extent to which the synaptic device can be modulated linearly in response to these stimuli. Both *β* and *N*_eff_ serve as important indicators of the complex response mechanisms exhibited by synaptic devices under external influences. Typically, a lower *β* value correlates with a higher *N*_eff_, thereby enhancing the device's capacity to modulate synaptic weights effectively in response to external stimuli. This modulation is crucial for achieving more accurate computational outcomes and improved learning capabilities within neural network applications. In this work, we conducted a nonlinear analysis on our ONS device, based on the fitting parameters extracted from the following weight update Equations (2) and (3):

(2) $$G_{n+1}=G_{n}+\Delta G_{P}=G_{n}+\alpha_{P}e^{-\beta_{P}\frac{G_{n}-G_{\min}}{G_{\max}-G_{\min}}}$$

(3) $$G_{n+1}=G_{n}+\Delta G_{D}=G_{n}-\alpha_{D}e^{-\beta_{D}\frac{G_{\max}-G_{n}}{G_{\max}-G_{\min}}}$$

where *G*_n+1_ and *G*_n_ represent the conductance of the device when the n+1*^th^* and n*^th^* pulse are applied, respectively. The maximum and minimum conductance values of the device are expressed as *G*_max_ and *G*_min_, respectively. Parameters *α*_P_ (for potentiation) and *α*_D_ (for depression) represent the changing step sizes of the conductance, while *β*_P_ (for potentiation) and *β*_D_ (for depression) represent the nonlinearity magnitude, for the device's LTP and LTD synaptic characteristics, respectively. Additionally, we define *N*_eff_ as the set of states in which Δ*G*_P,D_ exceeds a specified percentage of |*G_max_ – G_min_* |, termed as the threshold_ΔG_, set at 0.7% in this study (i.e., threshold_ΔG_ = 0.7% · |*G_max_* − *G_min_* |). Using threshold_ΔG_ as a criterion helps exclude pulse signals that produce states with low Δ*G*_*P*, *D*_ values, which are insignificant for neuromorphic computing, thereby reducing power consumption during computation. The fitting results for both LTP and LTD characteristics of our ONS device, according to the spectral wavelengths of light stimuli and frequency of input pulses, are summarized in Supporting Information Table S1 (Supporting Information). Our findings confirm that a faster frequency of input pulses leads to smaller nonlinearity, regardless of the spectral wavelengths of the light stimuli.

### Measurement Details

To construct the energy band structure of the ONS device, ultraviolet photoelectron spectroscopy (PHI VersaProbe 4) was employed. The absorption spectrum of the CsPbBr_3_ QDs was obtained using a Newport xenon light source, a monochromator (CS130B), and a power meter (1918‐R), which were integrated with an integrating sphere. Electroluminescence spectra and intensities of the fabricated samples were measured with a portable spectrometer (Ocean Optics USB4000). All electrical measurements of the fabricated devices were conducted using a source meter (Keithley 2400). The illuminated light sources were light‐emitting diodes emitting at wavelengths of 325 nm (ultraviolet), 420 nm (blue), and 525 nm (green), with the optical power varying between 0.05 and 3.00 mW. Optical pulses were modulated as the presynaptic stimulus for the ONS device using a waveform signal generator (Cleqee JDS6600). The electrical signals corresponding to the postsynaptic response of the ONS device were amplified using a low‐noise current preamplifier (Stanford Research Systems SR570) and subsequently analyzed with an oscilloscope (Keysight DSOX1204G). Transient measurements of the ONS device were performed utilizing a function generator (Agilent 33220A), an oscilloscope (Keysight DSOX1204G), and a benchtop optical power meter (Newport 1936‐R) paired with a photodiode (Newport 818‐UV/DB) covering the spectral range of 200–1100 nm.

## Conflict of Interest

The authors declare no conflict of interest.

## Author Contributions

Y.C.Y. and C.J.L. contributed equally to this work. C.S.L., J.K.S, and Y.J.L. conceived and initiated the work. Y.C.Y., C.J.L., and Y.J.L. designed the experiments. Y.C.Y., Y.J.C., and J.Z.F. fabricated the devices and performed the experiments. Y.C.Y., C.J.L., and H.O. performed simulations of colored image recognition. Y.C.Y., C.J.L., and Y.J.L. drafted the manuscript with input from other authors. C.S.L., J.K.S, and Y.J.L. supervised this work. All authors discussed the results and commented on the manuscript.

## Supporting information



Supporting Information

## Data Availability

The data that support the findings of this study are available from the corresponding author upon reasonable request.
