# Supplementary material for: All‐Inorganic Perovskite Quantum‐Dot Optical Neuromorphic Synapses for Near‐Sensor Colored Image Recognition
Source: Adv Sci (Weinh). 2024 Dec 16;12(5):2409933. doi: 10.1002/advs.202409933 (PMC11791932; doi:10.1002/advs.202409933)
Supplement: Supplementary file 1 — Supporting Information [file ADVS-12-2409933-s001.docx]

Supporting Information

All-Inorganic Perovskite Quantum-Dot Optical Neuromorphic Synapses for Near-Sensor Colored Image Recognition

Yung-Chi Yao, Chia-Jung Lee, Yong-Jun Chen, Jun-Zhi Feng, Hongseok Oh, Chin-Shan Lue*, Jinn-Kong Sheu*, Ya-Ju Lee*

Y.-C.Yao, C.-J. Lee, Y.-J. Chen, C.-S. Lue, J.-K. Sheu, Y.-J. Lee

Program on Key Materials, Academy of Innovative Semiconductor and Sustainable Manufacturing (AISSM), National Cheng Kung University,

No. 1, University Road, Tainan City 70101, Taiwan
E-mail: [cslue@mail.ncku.edu.tw](mailto:cslue@mail.ncku.edu.tw); [jksheu@ncku.edu.tw](mailto:jksheu@ncku.edu.tw); [yjlee@gs.ncku.edu.tw](mailto:yjlee@gs.ncku.edu.tw)

J.-Z. Feng, J.-K. Sheu, Y.-J. Lee

Department of Photonics, National Cheng Kung University,

No. 1, University Road, Tainan City 70101, Taiwan

H. Oh

Department of Physics, and Department of Intelligent Semiconductors, Soongsil University,

369 Sangdo-ro, Dongjak District, Seoul 06978, South Korea

C.-S. Lue

Department of Physics, National Cheng Kung University,

No. 1, University Road, Tainan City 70101, Taiwan

**Supplementary Figure 1**


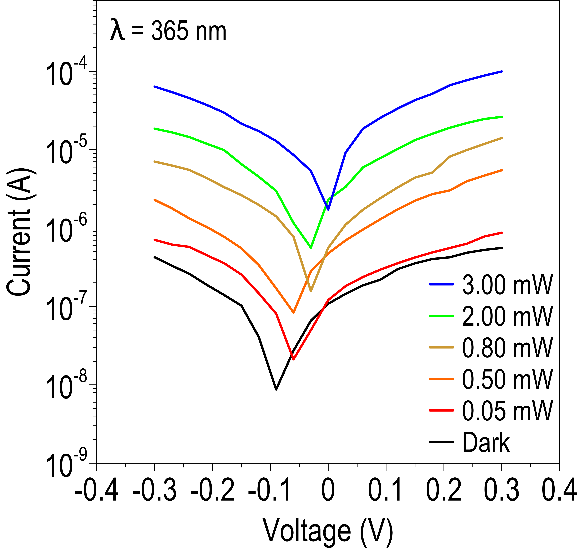


**Figure S1**. Current-voltage (*I-V*) characteristics of the CsPbBr_3_ QD-based p-i-n homojunction photodetector (the left unit of the ONS device) under UV-light illuminations (*λ* = 365 nm) with optical powers ranging from 0.05 to 3.00 mW. For comparison, the *I-V* curve of the device under the dark ambient condition is also included in the figure.

**Figure S1** presents the variations of resistance states observed in our CsPbBr_3_ QD-based p-i-n homojunction photodetector (i.e., the left unit of the ONS device) when exposed to UV-light illumination (*λ* = 365 nm) with optical power ranging from 0.05 to 3.00 mW. The data reveal a progressive increase in photocurrent in response to the incremental optical power. Specifically, under UV-light illumination with an optical power of 3.00 mW, the photocurrent of the device increases by approximately two orders of magnitude compared to the dark ambient condition, underscoring the device's high sensitivity and efficiency. Furthermore, it is important to ensure the photocurrent generated by the CsPbBr_3_ QD-based p-i-n homojunction photodetector under illuminations matches the HRS current of the RRAM on the right unit. Significant discrepancies in resistance values between left and right units would render adjustments in illumination ineffective for modifying the synaptic dynamics of the ONS device. Therefore, in this work, an incident optical power of UV-light illumination exceeding 0.8 mW is required to achieve proper resistance matching and effectively adjust the ONS device's synaptic dynamics. However, in some instances, we also employ an incident power of 0.5 mW with extended illumination durations to facilitate the optical coding process in the ONS device, as the *I-V* characteristics of our perovskite photodetector measured at both 0.5 mW and 0.8 mW are quite similar.

**Supplementary Figure 2**


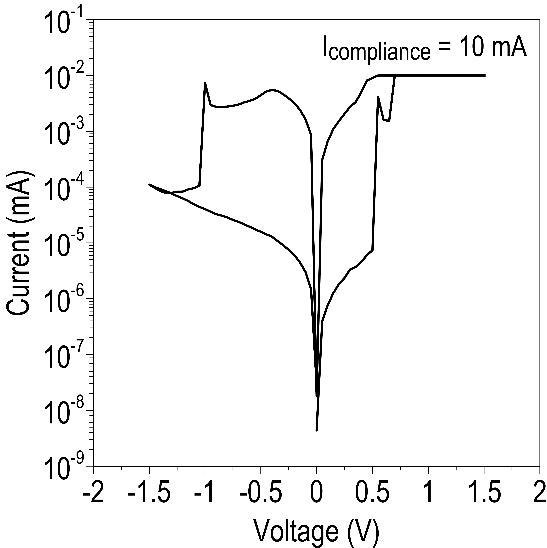


**Figure S2**. *I-V* characteristic of the CsPbBr_3_ QD-based RRAM (the right unit of the ONS device) under the dark ambient condition.

**Figure S2** depicts a representative *I-V* characteristic of the CsPbBr_3_ QD-based RRAM (i.e., the right unit of the ONS device) under the dark ambient condition. The measurement of the *I-V* curve involves a cyclic sweep across positive and negative voltages (0 V → 1.5 V → 0 V → –1.5 V → 0 V) with a current compliance of 10 mA. The set and reset voltages are observed at around 0.5 V and –1.0 V, respectively. During positive voltage scanning, the RRAM demonstrates a substantial expansion of its memory window by approximately three orders of magnitude, exhibiting a rapid transition from a high-resistance state (HRS) to a low-resistance state (LRS), confirming the reliable memory storage capability. Conversely, negative voltage sweeps promptly revert the LRS back to the HRS. Of critical importance for effective modulation in the synaptic dynamics of our ONS device is ensuring that the photocurrent generated by the CsPbBr_3_ QD-based p-i-n homojunction photodetector on the left unit matches or exceeds the HRS current (10^-6^ – 10^-5^ A) of the RRAM on the right unit. As discussed earlier in reference to **Figure** **S1**, this necessitates limiting the incident optical power of UV-light illumination to no less than 0.8 mW.

**Supplementary Figure 3**


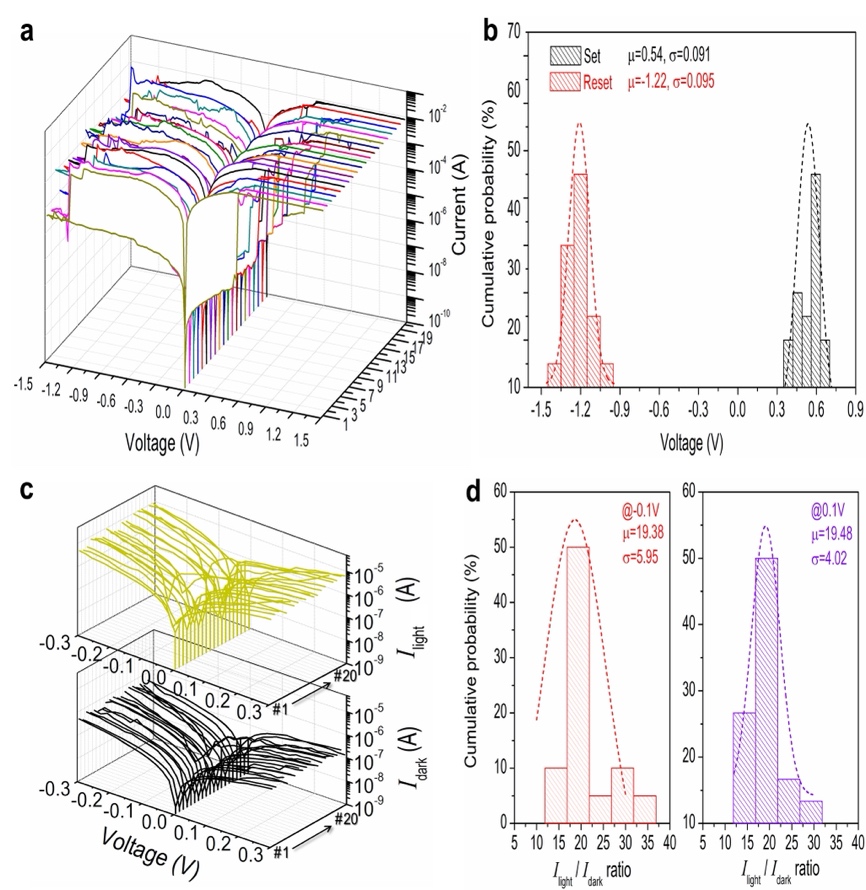


**Figure S3**. **a)** *I-V* characteristics of 20 CsPbBr3 QD-based RRAM devices on the right unit of the ONS device, with a compliance current set to *I_CC_* = 10 mA. **b)** Cumulative probability distributions of the set (right) and reset (left) voltages. **c)** *I-V* characteristics of 20 CsPbBr3 QD-based photodetector devices on the left unit of the ONS device under UV-light illumination (*λ* = 365 nm), optical power = 0.8 mW (bottom panel). *I-V* characteristics of these devices in dark ambient conditions are also included for comparison, (bottom panel). **d)** Cumulative probability distributions of the ratio between photocurrent and dark current, measured at +0.1 V (right) and –0.1 V (left) applied to the device.

**Figure S3a** shows the *I-V* characteristics of 20 CsPbBr_3_ QD-based RRAM devices on the right unit of the ONS, illustrating the variability in set and reset voltages among devices. **Figure S3b** presents the cumulative probability distribution of these set and reset voltages, based on the results derived from the devices shown in **Figure S3a**. The set voltage is centered around a mean of μ = 0.54 V with a small standard deviation of σ = 0.091, while the reset voltage has a similar distribution, with a mean of μ = –1.22 V and a slightly larger standard deviation of σ = 0.095. Additionally, **Figure S3c** shows *I-V* characteristic variations across 20 CsPbBr_3_ QD-based photodetector devices on the right unit of the ONS, for both with (top) and without (bottom) UV-light illuminations. The ratio of light-induced photocurrent under illumination ($I_{light}$) to dark current ($I_{dark}$), measured at ±0.1 V, is also calculated, and the corresponding cumulative probability distributions are displayed in **Figure S3d**. The $I_{light}/I_{dark}$ ratio at +0.1 V is centered around a mean of μ = 19.48 with a standard deviation of σ = 4.02. In comparison, the ratio slightly decreases with a mean of μ = 19.38 and a larger standard deviation of σ = 5.95. The above observations validate that our CsPbBr_3_ QD-based ONS device exhibits an acceptable device-to-device variability.

**Supplementary Figure 4**


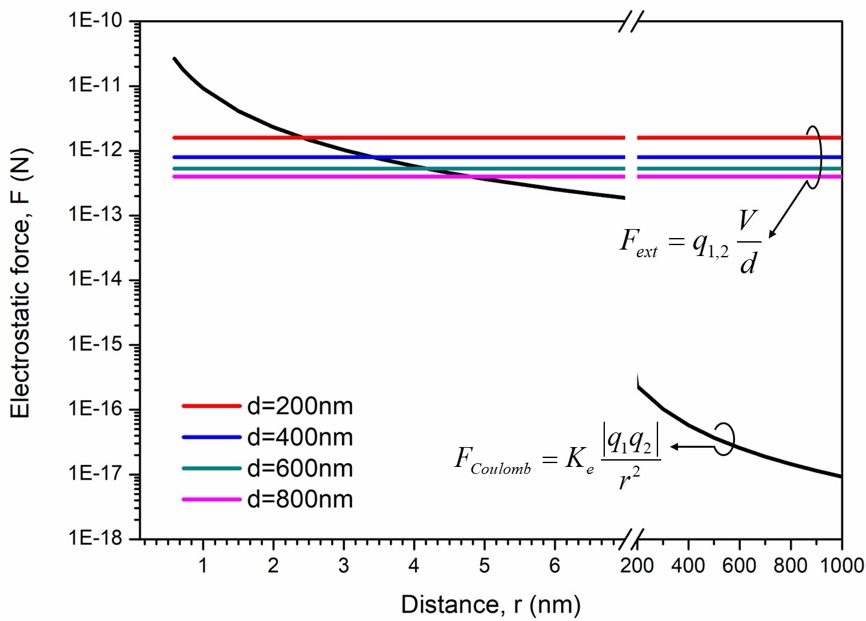


**Figure S4**. Estimated Coulomb attraction/repulsion force ($F_{Coulomb}$) acting on the charged ions within the perovskite material of the ONS device as a function of their separation distance ($r$). The forces experienced by charged ions due to the applied electrical bias ($F_{ext}$) are also plotted in the figure for different device thicknesses.

When an electrical bias ($V$) is applied to the ONS device, it creates an electric field similar to that of the parallel plate capacitor, expressed as $E=V/d$, where $d$ is the device thickness. Consequently, cations and anions such as Cs^+^, Br^–^, Ag^+^, and V_Br_^+^, are generated within the perovskite material and experience a force given by:

$F_{ext}=q_{1,2}\frac{V}{d}$ (S1)

Here $q_{1}$ and $q_{2}$ represent the charge quantity of cation and anion, respectively. In addition, these charged ions interact with each other through attraction or repulsion according to Coulomb’s law:

$F_{Coulomb}=K_{e}\frac{\left| q_{1}q_{2} \right|}{r^{2}}=K_{e}\frac{\left| q_{1} \right|^{2}}{r^{2}}=K_{e}\frac{\left| q_{2} \right|^{2}}{r^{2}}$ (S2)

where $K_{e}$ is a factor related to the dielectric constant of perovskite materials, and $r$ is the distance between ions. **Figure S4** depicts the estimated Coulomb attraction/repulsion force acting on the charged ions within the perovskite material of the ONS device as a function of $r$. The forces experienced by charged ions due to the applied electrical bias are also plotted in the figure for different device thicknesses ranging from $d=200$ to $800$ nm. Regardless of the device thickness, the magnitudes of $F_{ext}$ and $F_{Coulomb}$ acting on the charged ions are comparable only when $r$ is small, specifically in the range of approximately 2 nm to 5 nm. Beyond this region, $F_{ext}$ becomes the dominant force that drives ion migrations, exceeding $F_{Coulomb}$ by several orders of magnitude. Therefore, while Coulomb attraction and repulsion forces continue to act on the charged ions within the perovskite material, they can be neglected compared to the forces induced by the electrical bias on a macroscopic scale.

**Supplementary Figure 5**


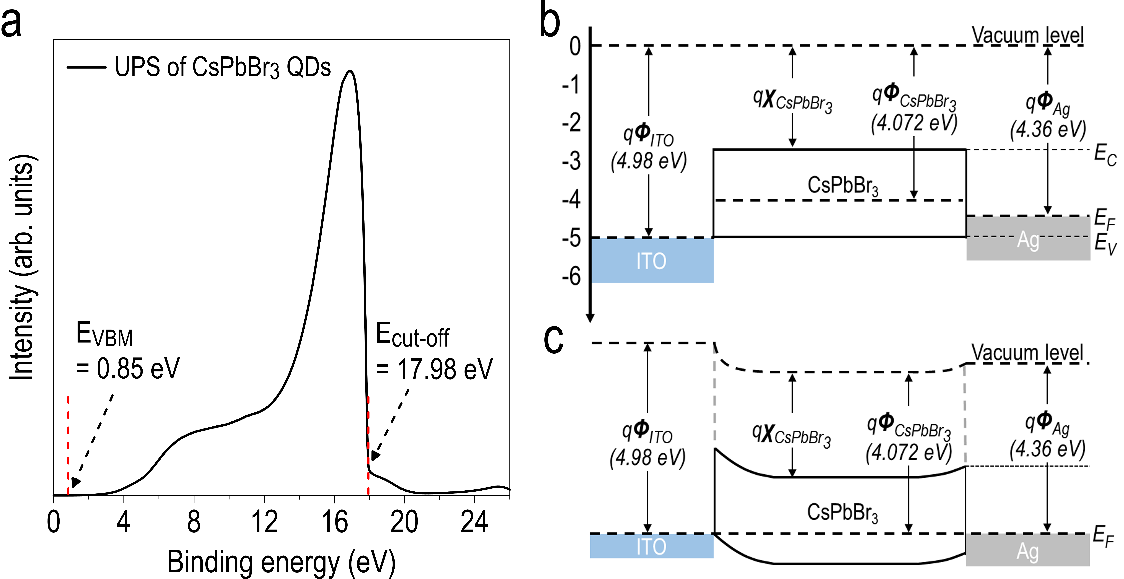


**Figure S5**. **a)** Ultraviolet Photoelectron Spectroscopy (UPS) curve of the CsPbBr_3_ QDs. Energy band diagram of the ITO/ CsPbBr_3_ QDs/Ag stack **b)** before and **c)** after thermal equilibrium.

To elucidate the operational mechanism of the ONS device, ultraviolet photoelectron spectroscopy (UPS) was employed to investigate the band structure of the CsPbBr_3_ QDs, as depicted in **Figure** **S5a**. The work function of the CsPbBr_3_ QDs (*WF_QD_*) was estimated through the following equation:

${WF}_{QD}=h\nu-(E_{cut-off}-E_{VBM})$ (S3)

where *hν* represents the ultraviolet incident energy at 21.20 eV, and $E_{cut-off}$ and *E_VBM_* extracted from **Figure** **S5a** are 17.98 eV and 0.85 eV, respectively. From **Equation S3**, the calculated *WF_QD_* relative to the vacuum energy level is approximately 4.07 eV. Consequently, the valence band maximum, given by *E_V_* = *WF_QD_* + *E_VBM_*, can be determined as 4.92 eV. Since the bandgap of the CsPbBr_3_ QDs (i.e., *E_g_* ≈ 2.36 eV) can be derived from its absorption spectrum (see **Fig.** **S7**), the conduction band minimum can hence be determined by *E_C_* = *E_V_* – *E_g_* ≈ 2.56 eV. Based on these numerical analyses, our CsPbBr_3_ QDs exhibit the p-type semiconductor behavior. Similarly, the work functions of Ag and ITO electrodes can also be determined by UPS to be *WF_Ag_* = 4.36 eV and *WF_ITO_* = 4.98 eV, respectively. **Figures** **S5b** and **S5c** illustrate the energy band diagram of the ITO/CsPbBr_3_ QDs/Ag stack both prior to and subsequent to thermal equilibrium, respectively. These diagrams are based on values derived from the UPS measurements depicted in **Figure** **S5a**, where dashed lines indicate the Fermi energy level. Due to the high work function of the Ag and ITO electrodes, the ohmic contact is established at the interfaces between the ITO/CsPbBr_3_ QDs/Ag stack, as substantiated by experimental evidence. Additionally, the small energy barrier between the ITO electrode and the CsPbBr_3_ QDs, and the Ag electrode and the CsPbBr_3_ QDs, facilitates the collection of photogenerated carriers when a positive electrical polarity is applied to the ONS device (see **Figure** **1b**), thereby enhancing photodetection performances of the CsPbBr_3_ QD-based p-i-n homojunction.

**Supplementary Figure 6**


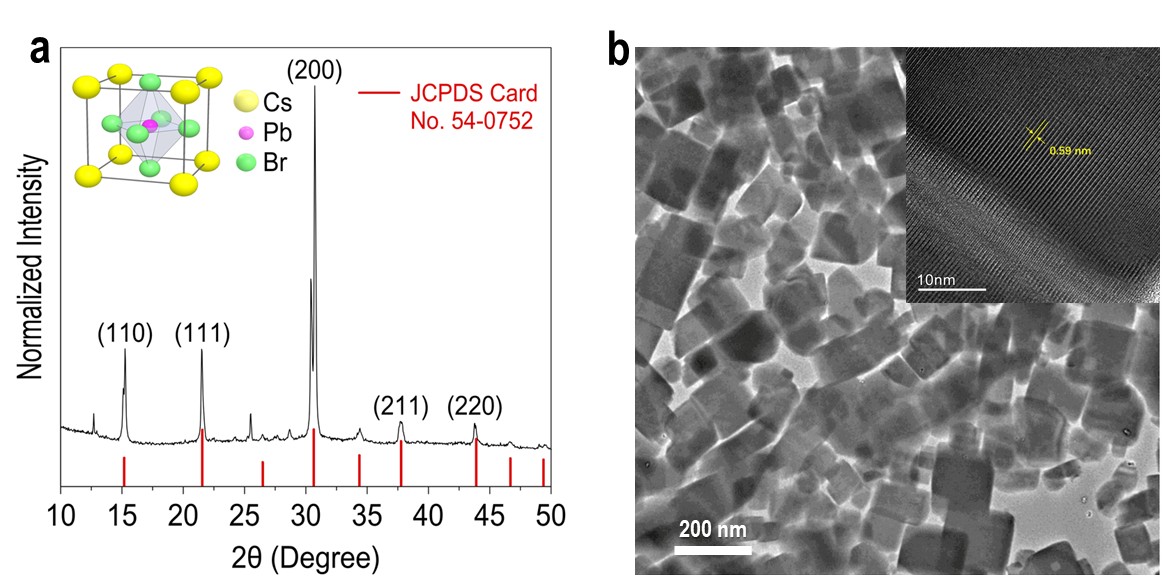


**Figure** **S6**. Morphology, crystallinity, and homogeneity characteristics of the synthesized CsPbBr_3_ QDs. **a)** XRD pattern and **b)** TEM image of the synthesized CsPbBr_3_ QDs using the supersaturated recrystallization method. 3D crystal structure of cubic CsPbBr_3_ and high-resolution TEM image are also inserted in **Figures** **S6a** and **S6b**, respectively.

**Figure** **S6a** shows the X-ray diffraction (XRD) analysis of our synthesized CsPbBr_3_ QDs. The analysis reveals a monoclinic crystal phase, which is similar to the cubic structure (JSPDS No. 54-0752) and can be identified by the split of the diffraction peak at approximately 31° corresponding to the (200) plane reflection. This indicates the high crystallographic quality and successful synthesis of our CsPbBr_3_ QDs. An inset in the figure provides a structural overview of CsPbBr_3_ in its cubic phase, illustrating a 3D framework of corner-connected octahedra with Cs^+^ ions occupying the cuboctahedral cavities. The morphology and homogeneity of the CsPbBr_3_ QDs were further examined using transmission electron microscopy (TEM), as shown in **Figure** **S6b**. During the supersaturated recrystallization process, the rapid transfer of Cs^+^, Pb2^+,^ and Br^–^ ions from a soluble solvent to an insoluble one significantly accelerates the growth of the QDs, leading to CsPbBr_3_ QDs with diameters of approximately 50–100 nm, much larger than the typical 10–15 nm size achieved by the hot-injection method. As a result, the synthesized QDs tend to aggregate, with distinct fusion interfaces forming between adjacent QDs. However, high-resolution TEM (HR-TEM) images indicate the highly crystalline nature of the cubic phase CsPbBr_3_, showing a d-spacing of 5.9 Å (inset, **Figure** **S6b**). This spacing corresponds to the distance between the (200) planes, as indexed in the XRD measurement.

**Supplementary Figure 7**


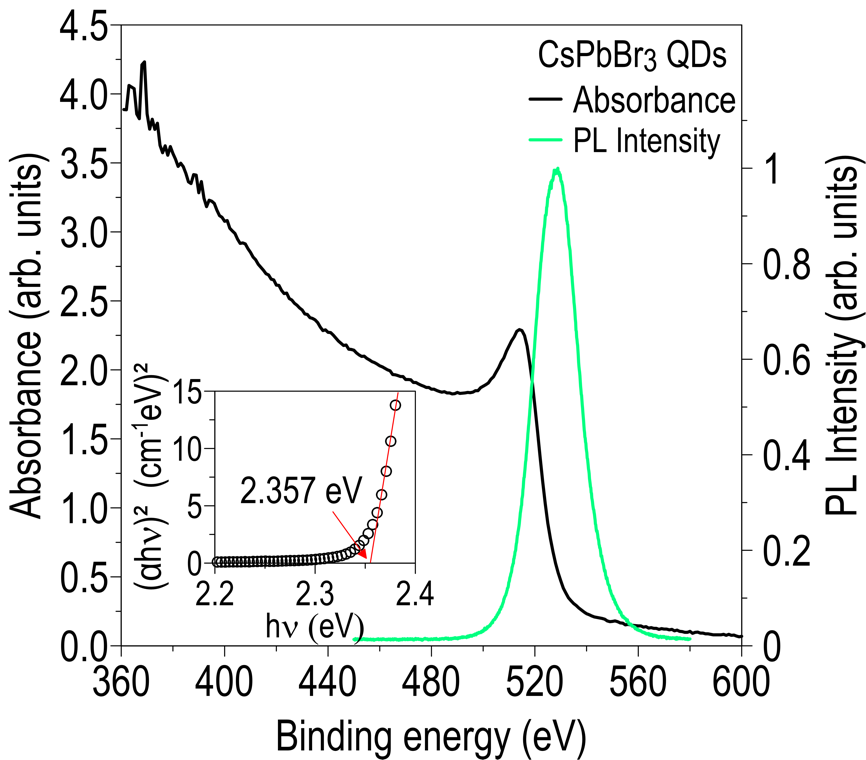


**Figure** **S7**. Absorption (primary vertical axis) and photoluminescence (*PL*, secondary vertical axis) spectra of the CsPbBr_3_ QDs synthesized through supersaturated recrystallization. Inset: square of optical absorption versus photon energy.

**Figure** **S7** shows the absorption (primary vertical axis) and photoluminescence (*PL*, secondary vertical axis) spectra of CsPbBr_3_ QDs synthesized through supersaturated recrystallization, utilized as the active layer of the ONS device. The CsPbBr_3_ QDs were spin-coated onto the sapphire substrate, and the measured absorption profile was calibrated against that of the bare sapphire substrate. The inset plots the square of the optical absorption as a function of photon energy, where the band gap of the CsPbBr_3_ QDs can be deduced to be around 2.357 eV by extrapolating the absorption to zero. Under excitation with a 405 nm laser diode, the CsPbBr_3_ QDs exhibit a sharp emission peak at λ = 526 nm. The absorption spectrum features a prominent excitonic peak at λ = 518 nm, which is about 8 nm Stokes-shifted from the *PL* emission, and shows a significant increase in optical absorption at shorter wavelengths. Quantitatively, the absorbance under UV-light illumination at 365 nm is approximately 1.5 times higher than that observed under blue illumination at 420 nm, and nearly 8 times higher than that under green illumination at 525 nm. This enhanced optical absorption at shorter wavelengths significantly boosts the generation of photocurrents within the p-i-n homojunction on the left unit of the ONS device. Consequently, this process facilitates the formation of conductive filaments in the RRAM on the right unit. The modulation of synaptic dynamics in the ONS device can be effectively achieved by selecting different optical wavelengths for illumination.

**Supplementary Figure 8**


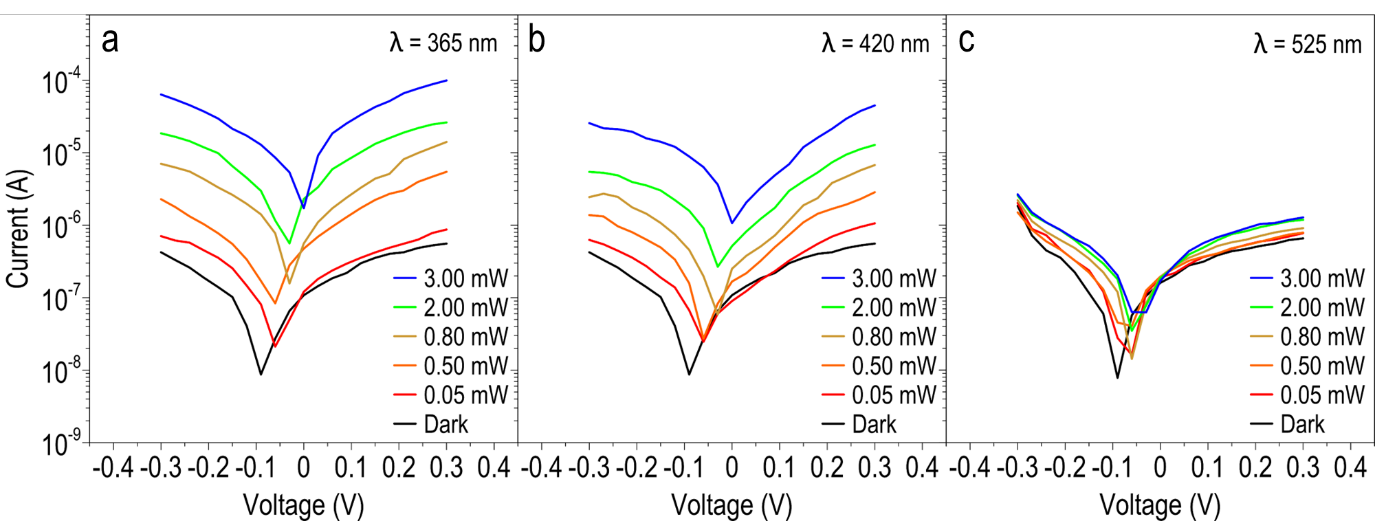


**Figure** **S8**. Comparative analysis of the variations in the *I-V* characteristics of the CsPbBr_3_ QD-based p-i-n homojunction photodetector under various wavelength illuminations. *I-V* characteristics of the CsPbBr_3_ QD-based p-i-n homojunction photodetector under illumination at three distinct wavelengths of λ = **a)** 365 nm, **b)** 420 nm, and **c)** 525 nm.

**Figure** **S8** provides a detailed comparative analysis of the variations in the *I-V* characteristics of the CsPbBr_3_ QD-based p-i-n homojunction photodetector when subjected to illumination at three distinct wavelengths of λ = **a)** 365 nm, **b)** 420 nm, and **c)** 525 nm. For each wavelength, the optical power was incrementally increased from 0.05 mW to 3 mW. In addition, the *I-V* characteristics of the device were also recorded under dark ambient conditions to establish a reference baseline. At an applied voltage of –0.3 V, the device exhibits a significant enhancement in photocurrent under UV illumination (λ = 365 nm), exceeding two orders of magnitude compared to the dark ambient conditions. Under blue illumination (λ = 420 nm), the enhancement in photocurrent is slightly reduced, approaching two orders of magnitude. Under green illumination (λ = 525 nm), the enhancement was further diminished, amounting to less than one order of magnitude. These findings suggest a pronounced accumulation of photogenerated electrons within the ITO layer under UV illumination, corroborating the absorption spectrum analysis presented in **Figure** **S7**. Consequently, in this study, an optical power of 0.8 mW was selected across all three wavelengths to trigger the synaptic dynamics of the ONS device. This selection was based on the observation that the resultant photocurrent of the p-i-n homojunction photodetector at this optical power level was comparable to the high resistance state (HRS) current (~5.0×10^-6^ A) of the adjacent RRAM device, as depicted in **Figure** **S2**.

**Supplementary Figure 9**


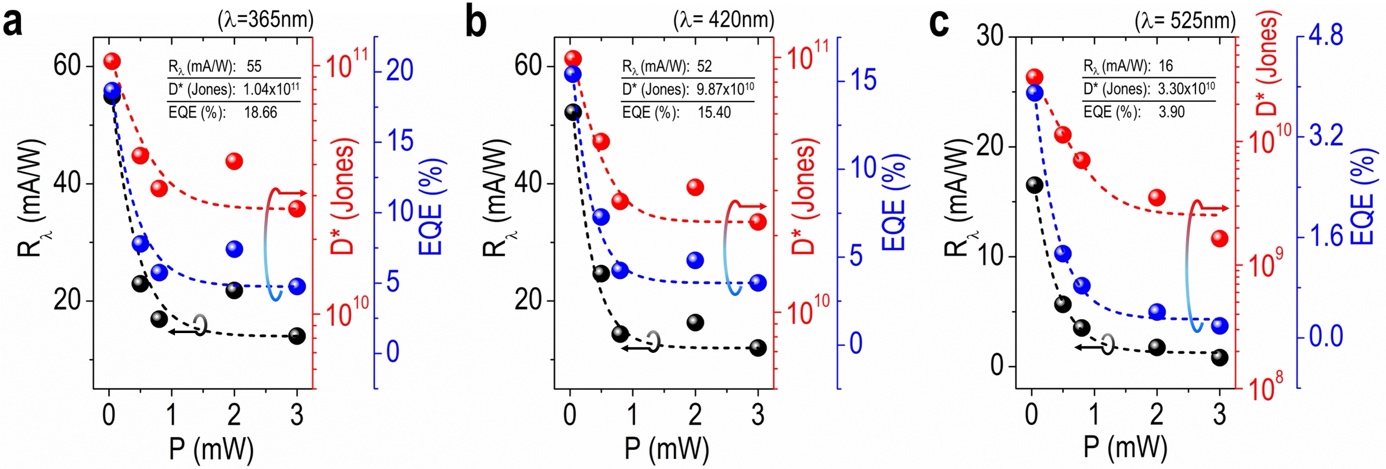


**Figure S9**. Dependence of responsivity ($R_{\lambda}$), detectivity ($D^{*}$), and external quantum efficiency ($EQE$) on light power ($P$) for the CsPbBr_3_ QD-based p-i-n homojunction photodetector, illuminated at three wavelengths: **a)** 365 nm, **b)** 420 nm, and **c)** 525 nm, with a forward bias of +0.3 V applied to the device. The maximum values of $R_{\lambda}$, $D^{*}$, and $EQE$, obtained at $P=0.01$ mW for each wavelength, are also summarized in the figure.

**Figure S9** presents a detailed analysis of the photoresponse performance of the CsPbBr_3_ QD-based p-i-n homojunction photodetector, highlighting key metrics such as responsivity ($R_{\lambda}$), detectivity ($D^{*}$), and external quantum efficiency ($EQE$). These metrics were measured over a power range of $P=0.01-3.0$ mW, under illumination at three spectral wavelengths of $\lambda=$ **a)** 365 nm, **b)** 420 nm, and **c)** 525 nm. Responsivity, which measures the photodetector's efficiency in converting incident light into photocurrent, is defined as the ratio of generated photocurrent (${\Delta I}_{ph}$) to incident light power ($P)$, and is given by:

$R_{\lambda}=\frac{{\Delta I}_{ph}}{P}=\frac{(I_{light}-I_{dark})}{P}$ (S4)

where $I_{light}$ and $I_{dark}$ represent the light-induced photocurrent under illumination and the dark current, respectively. Across all wavelengths, $R_{\lambda}$decreases as $P$ increases due to saturation effects at higher power levels, with the highest $R_{\lambda}$consistently observed at the lowest power, $P=0.01$ mW. At any given power level, $R_{\lambda}$ decreases as $\lambda$ increases, a trend consistent with the absorption properties of CsPbBr_3_ QDs shown in **Fig. S7**. The maximum responsivity achieved in this study was $R_{\lambda}=$55 mA/W, 52 mA/W, and 16 mA/W for $\lambda=$ 365 nm, 420 nm, and 525 nm, respectively, which is consistent with the previous studies on CsPbBr_3_-based photodetectors.^[1-4]^

Detectivity, which indicates the minimum detectable level of incident light, is strongly influenced by the dark current and is expressed as:

$D^{*}=\frac{R_{\lambda}}{\sqrt{2qJ_{dark}}}$ (S5)

where $J_{dark}$ is the dark current density and $q$ is the elementary charge. As $P$ increases, $D^{*}$follows a similar decreasing trend, driven by the behavior of $R_{\lambda}$ in the numerator of **Equation S5** and the consistently low dark current in the sample. The highest detectivity achieved was $D^{*}=1.1\times{10}^{11}$ at $\lambda=$ 365 nm, decreasing slightly to $D^{*}=9.87\times{10}^{10}$ Jones and $D^{*}=3.30\times{10}^{10}$ Jones for $\lambda=$ 420 nm and 525 nm, respectively.

Finally, the external quantum efficiency ($EQE$), which quantifies the fraction of incident photons that generate external photocurrent, is defined as:

$EQE=\frac{hc}{q\lambda}\cdot$ $R_{\lambda}$ (S6)

where $h$ is Planck’s constant and $c$ is the speed of light in a vacuum. The maximum $EQE$ values measured in this study were $EQE=18.66\%$, $15.40\%$, and 3.90% for $\lambda=$ 365 nm, 420 nm, and 525 nm, respectively.

1. Y. Dong, Y. Gu, Y. Zou, J. Song, L. Xu, J. Li, J. Xue, X. Li, H. Zeng, *Small* **2016**, 12, 5622− 5632.
2. J. Song, L. Xu, J. Li, J. Xue, Y. Dong, X. Li, H. Zeng, *Adv. Mater*. **2016**, 28, 4861−4869.
3. X. Li, D. Yu, F. Cao, Y. Gu, Y. Wei, Y. Wu, J. Song, H. Zeng, *Adv. Funct. Mater.* **2016**, 26, 5903−5912.
4. C. Zhao, Y. Liu, L. Chen, J. Li, H. Y. Fu, S. Zhao, W.-D. Li, G. Wei, *ACS Appl. Electron. Mater.* **2021**, 3, 337−343.

**Supplementary Figure 10**


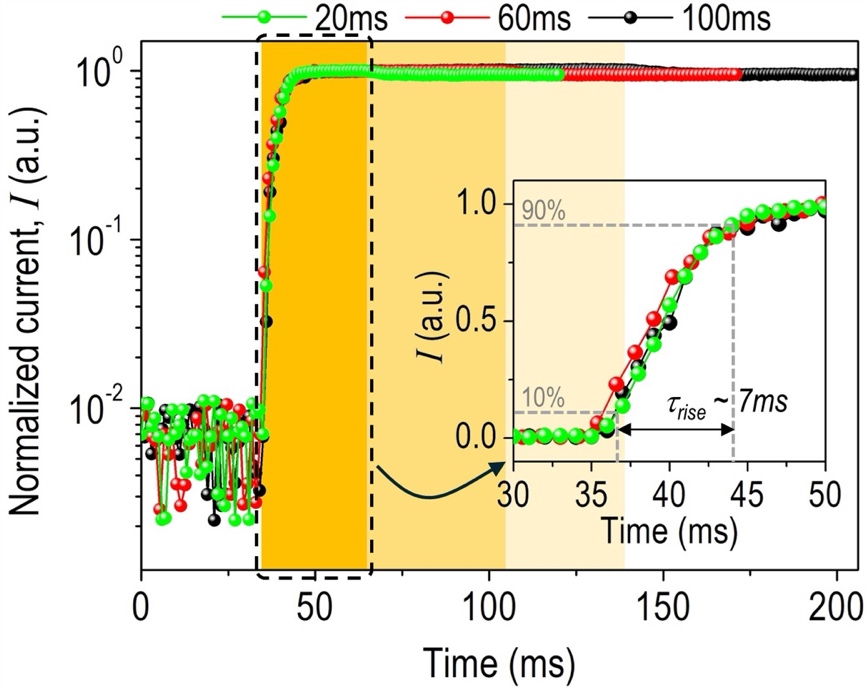


**Figure S10**. Temporal variations in the current of the ONS device when the left unit was exposed to UV-light for different durations of 100 ms, 60 ms, and 20 ms. Inset: the enlarged rising edge of the current, where $\tau_{rise}$, defined as the time difference between 10% and 90% of the maximum current, is estimated to be ~ 7 ms for all three exposure durations.

To determine the activation time required for the optical encoding process in our ONS device, we systematically shortened the duration of UV-light exposure on the left unit and monitored the resulting changes in current across the entire device. **Figure S10** illustrates the variation in current over time in the ONS device when the left unit was exposed to UV-light for different durations of 100 ms, 60 ms, and 20 ms. A constant bias of +1.5 V was applied to the ONS device, and the incident power of the UV light was kept at 0.5 mW during all measurements. In each case, the device's current significantly increased by approximately two orders of magnitude during UV illumination and remained elevated even after the UV-light was turned off. This confirms a successful transition of the ONS device from HRS to LRS. The figure also includes an enlarged view of the rising edge of the current, showing that the rise time ($\tau_{rise}$), defined as the time difference between reaching 10% and 90% of the maximum current, was consistently measured at around 7 ms for all three exposure durations. These results indicate that UV-light exposure must exceed 7 ms (or be less than 142 Hz) to effectively activate the optical encoding process in the ONS device.

**Supplementary Figure 11**


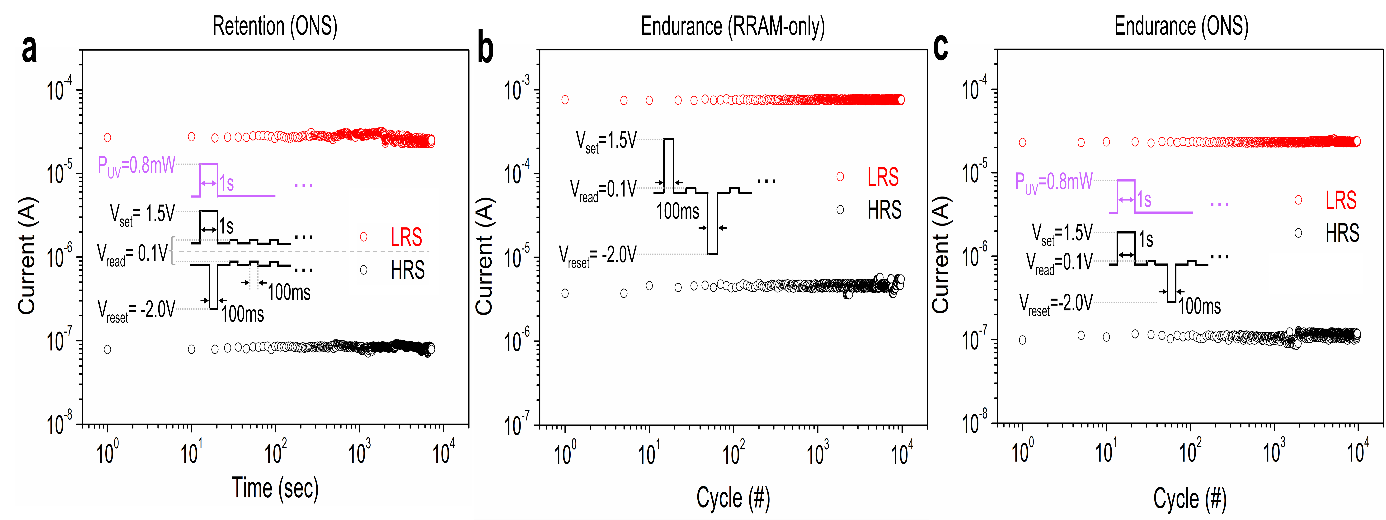


**Figure S11**. **a)** Retention performance of the LRS and HRS states in the RRAM device (the right unit of the ONS device). **b)** Endurance test results of the standalone RRAM device, read at 0.1 V, over 10^4^ cycles. **c)** Endurance test results of the ONS device, where the RRAM unit was set to the LRS state under the same conditions as in **Fig. S11a**, and then reset to the HRS state by applying −2.0 V for 100 ms. The corresponding electrical bias and optical pulse settings used for the retention and endurance tests are also inserted in the figures.

**Figure S11a** shows the retention performance of the perovskite RRAM on the right unit of the ONS device. To set the RRAM unit to the LRS state, a pulse bias of +1.5 V was applied across the ONS device for 1 second, while the left unit of the ONS device was simultaneously exposed to UV light (λ = 365 nm) at an incident power of 0.8 mW for the same duration. The RRAM unit was then reset to the HRS state by applying a −2.0 V bias for 100 ms. During the retention test, consecutive square-wave bias pulses (0.1 V, 100 ms pulse-width, and 1.0-second period) were used to monitor the HRS and LRS states. Despite minor fluctuations, both states remained stable for over 10^4^ seconds, with an ON/OFF current ratio of approximately 10^2^, demonstrating excellent retention stability. **Figure S11b** presents endurance testing results for the standalone RRAM, which underwent a repetitive sequence of 1.5 V (Set) / 0.1 V (Read) / −2.0 V (Reset) / 0.1 V (Read), with each step lasting 100 ms. The HRS/LRS ratio of ~10^2^ shows no significant degradation even after more than 10^4^ sweeping cycles. **Figure S11c** evaluates the endurance of the ONS device under conditions similar to those in **Figure S11a**, where a +1.5 V pulse and UV illumination were used on the ONS device to set its RRAM unit to the LRS state, followed by a −2.0 V pulse applied on the ONS device for 100 ms to reset its RRAM unit to the HRS state. A 0.1 V pulse (100 ms duration) was applied to monitor the current switching between the LRS and HRS states. The ONS device maintains consistent switching behavior over 10^4^ sweeping cycles, with a stable HRS/LRS ratio above 10^2^. Overall, these results shown in **Figure S11** confirm the reliable and reproducible write/erase performance of the ONS device, whether activated electrically or optically.

**Supplementary Figure 12**


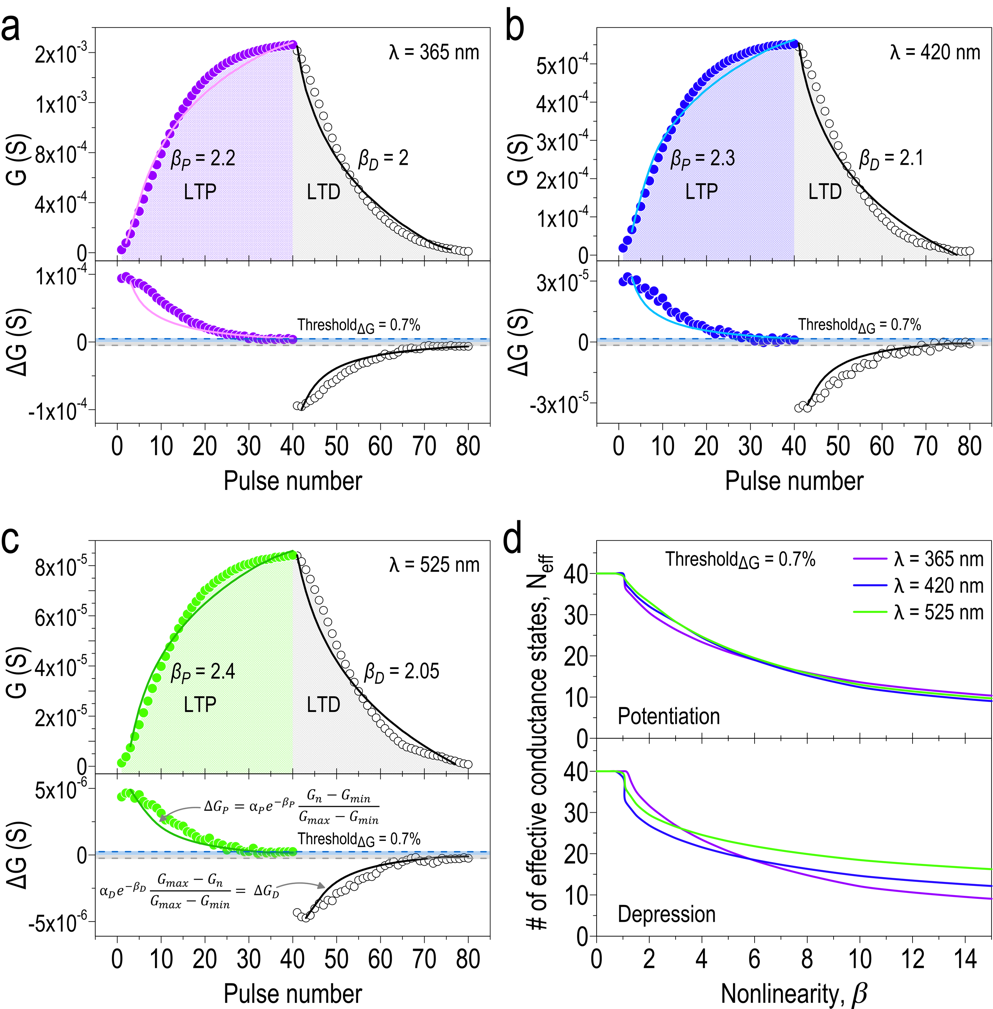


**Figure S12**. LTP and LTD curves (top panels) and their corresponding changes in two consecutive conductances (*∆G*, bottom panels) as a function of the number of applied pulses for different light wavelengths of **a)** 365 nm, **b)** 420 nm, and **c)** 525 nm. During the LTP process, the left unit of the ONS device is exposed to consecutive light pulses, each having an average optical power of 0.8 mW, a duration of 0.25 s, and a repetition rate of 2 Hz. For the LTD process, electrical pulses with the identical duration time and repetition rate as the optical pulses, but with an amplitude of –2.0 V, are applied to the right unit of the ONS device. **d)** The calculated number of effective conductance states as a function of nonlinearity for various light wavelengths, with a threshold_ΔG_ of 0.7%, for both LTP (top) and LTD (bottom) processes.

The performance of the artificial synaptic device is greatly affected by the nonlinearity in the LTP and LTD curves, which vary with the number of applied pulses. **Figure S12** shows the LTP and LTD curves (top panels), along with their corresponding changes in two consecutive conductances (*∆G*, bottom panels), as a function of the number of applied pulses at different light wavelengths of **a)** 365 nm, **b)** 420 nm, and **c)** 525 nm. While the LTP/LTD curves occupy different conductance ranges depending on the light wavelength, their overall shapes remain similar, and the nonlinearity values for both LTP (*β*_P_) and LTD (*β*_D_) are nearly identical, as calculated using equations (2) and (3) from the weight update equation illustrated in the Experimental Methods section. In general, high nonlinearity values require additional peripheral circuits to regulate the effective range of conductance changes, complicating the operation of the synaptic device.^[5,6]^

Regardless of the light wavelength, the bottom panels of **Figs. S12a-S12c** show that during the LTP and LTD processes, *∆G* decreases and increases exponentially, respectively, as the number of applied pulses rises, eventually reaching a saturation region (shaded in colors). In this region, further applied pulses have minimal impact on conductance changes, making them ineffective. The number of effective conductance states, *N_eff_*, is hence defined as the number of states where *∆G* exceeds a specified percentage of |*G_max_ – G_min_*|, termed as the threshold_ΔG_, which is set to 0.7% in this study (i.e., threshold_ΔG_ $=0.7\%\cdot\left| G_{max}-G_{min} \right|$). For instance, out of 40 ∆G points during the LTP process depicted in **Fig. S12a**, 9 fall below the threshold_ΔG_, resulting in *N*_eff_ being 31 out of 40. Following this approach, the number of effective conductance states for the ONS device was evaluated under different light wavelengths and pulse frequencies for both LTP and LTD processes, with the results summarized in Supporting Information **Table S1**. Accordingly, using 40 consecutive pulses effectively initiates and stimulates the cognitive dynamics of our ONS device, while applying more pulses would only produce nonlinear and saturated changes in conductance, unnecessarily increasing power consumption during computation. To further investigate the relationship between *N*_eff_ and *β*, we calculated the number of effective conductance states relative to the nonlinearity by maintaining a threshold_ΔG_ value of 0.7% for both LTP and LTD processes, as shown in **Fig. S12d**. The results confirm that regardless of light wavelength, as the conductance response becomes more nonlinear, the number of effective conductance states decreases exponentially, posing a challenge for effectively tuning the device's synaptic weights. Therefore, it is important to ensure that the fabricated synaptic device maintains acceptable nonlinearity characteristics.

1. R. Mochida et al., In *2018 Symposium on VLSI Technology* (IEEE, Honolulu, 2018)
2. S. Ambrogio et al., *Nature*. **2018**, 558, 60−67.

**Supplementary Table 1**

**Table S1.** Fitting parameters extracted from the weight update equation for colored image recognition.

| *λ*(nm) | *f* (Hz) | *α*_P_ | *α*_D_ | *β*_P_ | *β*_D_ | *G_max_* (S) | *G_min_* (S) | *N_eff, P_* | *N_eff, D_* |
| --- | --- | --- | --- | --- | --- | --- | --- | --- | --- |
| 365 | 1 | 4.01x10^-4^ | 5.49x10^-4^ | 4.0 | 4.0 | 1.27x10^-3^ | 1.26x10^-4^ | 22 | 23 |
| 365 | 2 | 1.36x10^-4^ | 1.68x10^-4^ | 2.2 | 2.0 | 1.66x10^-3^ | 2.38x10^-4^ | 31 | 32 |
| 365 | 4 | 6.79x10^-5^ | 9.01x10^-5^ | 1.0 | 1.1 | 1.78x10^-3^ | 3.60x10^-4^ | 40 | 40 |
| 420 | 1 | 1.18x10^-5^ | 1.20x10^-5^ | 4.4 | 3.9 | 3.80x10^-5^ | 1.54x10^-6^ | 23 | 21 |
| 420 | 2 | 4.79x10^-5^ | 5.52x10^-5^ | 2.3 | 2.1 | 5.26x10^-4^ | 6.64x10^-5^ | 32 | 27 |
| 420 | 4 | 2.41x10^-5^ | 2.40x10^-5^ | 1.1 | 1.0 | 5.55x10^-4^ | 2.59x10^-5^ | 40 | 40 |
| 525 | 1 | 2.49x10^-5^ | 1.52x10^-5^ | 4.5 | 4.0 | 5.56x10^-5^ | 1.50x10^-6^ | 24 | 25 |
| 525 | 2 | 8.21x10^-6^ | 8.01x10^-6^ | 2.4 | 2.1 | 8.42x10^-5^ | 7.41x10^-6^ | 33 | 29 |
| 525 | 4 | 3.68x10^-6^ | 4.02x10^-6^ | 1.0 | 1.0 | 9.82x10^-5^ | 7.68x10^-6^ | 40 | 40 |
